# The Contribution of Common and Rare Genetic Variation to Emotional and Behavioural Symptoms in Childhood and Adolescence

**DOI:** 10.1038/s41380-026-03851-6

**Published:** 2026-09-11

**Authors:** Olivia Wootton, Emma E. Wade, Daniel S. Malawsky, Mahmoud Koko, Wei Huang, Qin Qin Huang, Varun Warrier, Matthew E. Hurles, Hilary C. Martin

**Affiliations:** 1 Wellcome Sanger Institute, Hinxton, UK; 2 Bioinformatics Interdepartmental Program, UCLA, Los Angeles, CA, USA; 3 Department of Psychiatry, University of Cambridge, Cambridge, UK; 4 Department of Psychology, University of Cambridge, Cambridge, UK

## Abstract

Genetic factors influence vulnerability to common mental health conditions, but their role in early-life mental health remains understudied. We analysed genotype array (n=4,709-6,687) and exome sequence data (n=4,500-5,424) from the Millennium Cohort Study (MCS) and Avon Longitudinal Study of Parents and Children (ALSPAC) to assess the contribution of common variants and rare deleterious coding variants to internalising and externalising symptoms across development. In longitudinal analysis spanning ages 5-17 years, we identified several associations between common genetic variation, indexed by polygenic indices (PGIs), and both symptom domains that generally remained stable across development. Effect sizes were modest, with the largest estimates observed for PGIs for attention deficit hyperactivity disorder (ADHD) and externalising behaviour with externalising symptoms (β=0.13 to 0.18; p-adj<3.5×10^−29^). Evidence for direct genetic effects was strongest for externalising symptoms, including for associations with the ADHD and externalising behaviour PGIs. Concordant results were observed in the Born in Bradford cohort. A higher exome-wide burden of deleterious rare variants was associated with increased externalising and internalising symptoms (β=0.04 to 0.06, p-adj<0.03); within-family models indicated direct genetic effects on externalising in MCS (β=0.07; p<0.05, p-adj>0.05) and on internalising symptoms in ALSPAC (β=0.12, p-adj<0.02). Common and rare genetic variants contributed independently, jointly explaining 2% of the variance in internalising and 5-7% in externalising symptoms. This study shows that early-life mental health is influenced by both common and rare genetic variation, with several associations explained by direct genetic effects.

## Introduction

Mental health conditions that emerge in childhood and adolescence often persist into adulthood [1] and are associated with a range of adverse health and developmental outcomes [2–9]. Both genetic and environmental factors contribute to early-life emotional and behavioural difficulties [10]. Twin studies estimate the heritability of internalising disorders (anxiety and depressive disorders) to be 30-50%, [11, 12] whereas externalising disorders (ADHD, conduct disorder, and oppositional defiant disorder) have higher heritability estimates of 50-80% [10, 13].

Understanding how genetic variation influences mental health traits across development and how these influences evolve over time may provide a clearer picture of behavioural trajectories. Genetic variants carried by a child may influence their phenotype directly; these are referred to as direct genetic effects [14]. Additionally, as children share genetic variants with their parents, associations between a child’s genotype and phenotype may also arise indirectly via genetically-influenced parental characteristics (e.g., parenting behaviour), leading to indirect genetic effects [15]. However, parent-offspring genetic associations may also reflect familial confounding, including population stratification and assortative mating [15–18]. Recent studies have increasingly leveraged within-family designs to try to distinguish direct genetic effects from broader familial sources of genotype-phenotype association.

Studies investigating direct and indirect genetic effects on early emotional and behavioural traits have so far focused on common variants (typically minor allele frequency ≥ 1-5%). Two widely used approaches are variance-component methods, such as trio-GCTA [18] and PGI-based family models [15]. Variance-component methods use genetic similarity among relatives, estimated from pedigree structure or measured and imputed genome-wide single nucleotide polymorphisms (SNPs), to estimate the proportion of phenotypic variance attributable to direct and indirect genetic components [18]. PGI-based family models instead test whether measured polygenic liability for specific traits, indexed by SNPs included in the PGI and variants tagged through linkage disequilibrium, is associated with offspring phenotypes through direct or familial pathways. Parent–offspring PGI designs jointly model offspring and parental PGIs to estimate direct genetic effects and the effects of non-transmitted parental alleles, which capture indirect genetic effects and confounders. Related within-family PGI-based approaches use sibling- and twin-based designs to provide estimates of direct genetic effects by controlling for shared familial confounding [19, 20]. This methodological heterogeneity and the limited availability of large, well-powered family-based cohorts have contributed to mixed conclusions regarding the role of indirect effects on mental health traits.

For externalising traits (e.g., impulsivity, hyperactivity, and aggression), variance-component approaches have identified both direct effects and effects of non-transmitted parental alleles [21–26], whereas PGI-based studies have primarily reported evidence for direct genetic effects [19, 27–29]. Additionally, evidence on how these genetic influences may vary across development is sparse. Two recent PGI-based studies that reported a direct genetic effect of an adult externalising behaviour PGI on externalising symptoms found that the magnitude of the association varied across development - peaking during childhood in one study [29], and increasing from childhood to adolescence in the other [24].

Research on internalising traits (e.g. anxiety and depression) remains limited. Although there is evidence for both direct and indirect genetic associations with childhood internalising difficulties, existing research is based on data from the same cohort [22, 25, 30]. To our knowledge, only one study considers changes over time, finding that the variance explained by direct and indirect effects increases from childhood to adolescence [31]. Both replication in independent samples and additional longitudinal analyses are needed to clarify these associations. More broadly, recruitment into family-based genetic studies is frequently subject to participation biases [32, 33], an issue that extends to work on mental health symptoms, potentially limiting the generalisability of findings and introducing biases into genetic effect estimates [34, 35].

Common variants account for less than half of the total heritability estimated from twin studies for early emotional and behavioural traits [36–38]. It is unknown how much of this “missing heritability” is attributable to rare variants. Rare copy number variants (CNVs) associated with developmental disorders are known to influence childhood mental health in both population-based[39] and clinically-ascertained cohorts [40–42]. Deleterious sequence-level mutations, such as protein-truncating and damaging missense variants, also contribute to related traits, like cognitive ability and educational attainment [43, 44], and to mental health conditions in adulthood [43, 45, 46].

In this work, we leverage genotype array and new exome-sequence data from two primary birth cohorts (ALSPAC and MCS) to investigate genetic risk factors for childhood and adolescent mental health symptoms. We use a third independent birth cohort, Born in Bradford (BiB) for replication. Throughout, we use “common variants” to refer to those captured by genotype arrays and aggregated into polygenic indices; “rare variants” refer to low-frequency coding variants, including protein-truncating and damaging missense variants, identified through exome sequencing, with study-specific allele frequency thresholds (see Methods). First, we use longitudinal mixed effects modelling to evaluate associations between these measures of common and rare genetic variation and repeated measures of internalising and externalising symptoms. We estimate these associations using the full ‘population’ sample and within-families (i.e. adjusting for parental genetic scores) to evaluate the contribution of direct genetic effects, and test for changes in these associations over time. Next, we investigate whether common and rare genetic variation contribute independently to variation in internalising and externalising symptoms and quantify the relative importance of each genetic measure for prediction of these traits. By integrating common and rare variant analyses with within-family designs, we aim to refine understanding of the mechanisms underlying these traits and inform future research into early identification and prevention of mental disorders.

## Methods

### Study design and participants

#### Avon Longitudinal Study of Parents and Children (ALSPAC)

ALSPAC is a prospective birth cohort study investigating genetic and environmental influences on health-related outcomes across the lifespan [47]. The study has been described in detail previously [47–49]. ALSPAC enrolled 14,541 pregnant women in South West England with expected dates of delivery between 1st April 1991 and 31st December 1992. Enrolled children (N=13,988) and their parents were routinely followed up by questionnaires and clinic visits. The study collected biological samples, including DNA, as well as information on health-related measures and environmental exposures. DNA samples were obtained from most children and a subsample of parents. A detailed description of the cohort is provided in the Supplementary Methods.

The study website (http://www.bristol.ac.uk/alspac/researchers/our-data/) contains details of available data through a fully searchable data dictionary and variable search tool. Ethical approval for the study was obtained from the ALSPAC Ethics and Law Committee and local research ethics committees. Consent for biological samples has been collected in accordance with the Human Tissue Act (2004). Informed consent for the use of data collected via questionnaires and clinics was obtained from participants following the recommendations of the ALSPAC Ethics and Law Committee at the time.

#### Millennium Cohort Study (MCS)

MCS is a prospective, nationally representative, birth cohort following 18,827 children born in the United Kingdom between September 2000 and January 2002 [50, 51]. The study employed strategic sampling, intentionally oversampling participants from disadvantaged areas and ethnic minorities in the United Kingdom to maximise the inclusion of groups that are typically under-represented in research [52]. Seven sweeps of data collection have been conducted since enrollment at ages 9 months and 3, 5, 7, 11, 14, and 17 years. Extensive demographic, health, developmental, and socio economic data has been collected from children and their families [50]. DNA samples were collected at age 14 from 9,259 cohort members, 8,898 mothers and 5,179 fathers [53].

MCS has ethical approval from the National Health Service Research Ethics Committee and the National Research Ethics Service.

#### Born in Bradford (BiB)

BiB is a prospective birth cohort that recruited 12,453 pregnant women from Bradford, a city in the north of England, between 2007 and 2011 [54, 55]. At the time of recruitment, 41% of the cohort self-reported white British heritage, and 68% of children lived in the most deprived quintile of neighbourhoods in England and Wales. Subsamples of enrolled children have been followed up at various ages for specific substudies and routine health and education data linkage is available for most children.

Ethics approval for BiB was obtained from the National Health Service Health Research Authority Yorkshire and the Humber (Bradford Leeds) Research Ethics Committee reference: 16/YH/0320 and 16/YH/0062). Mothers were consented to participate in the study upon attendance of a routine antenatal clinic appointment at the city’s primary maternity unit.

### Phenotypic measures

#### Internalising and externalising symptoms

The Strengths and Difficulties Questionnaire (SDQ) was used to assess emotional and behavioural symptoms at several time points in all cohorts. The SDQ is a well-validated, 25-item questionnaire widely used to screen for mental health problems in community samples of children and adolescents [56]. It may be completed by parents and teachers of children and adolescents and, after the age of 11, by the children themselves. The questionnaire assesses five domains of behaviour: hyperactivity/inattention, conduct problems, peer relationship problems, emotional symptoms, and prosocial behaviour. A score for externalising symptoms is calculated by summing scores for the hyperactivity/inattention and conduct problems subscales, and an internalising symptoms score is calculated as the sum of the peer relationship problems and emotional symptoms subscales [57, 58]. This results in a maximum score of 20 for both the externalising and internalising scales, with higher scores reflecting an increased symptom burden.

For the main analyses, parent-reported SDQ scores from ages 5, 7, 11, 14, and 17 in MCS and ages 7, 9, 11, 13, and 16 in ALSPAC were used. The SDQ was completed by mothers in ALSPAC and by the main caregiver in MCS. For uniformity and to minimise potential reporter biases, the analysis was restricted to mother-reported SDQ scores. In BiB, parents completed the SDQ at ages 3, 4, 10, 11, and 12 years. As information on which parent completed the questionnaire was not available for BiB, all parent-reported scores were analysed together.

### Preparation of genotype data

Processing and quality control of the genotype array data are described in detail in the Supplementary Methods. Briefly, initial sample-level quality control removed heterozygosity outliers, sex mismatches, and individuals exceeding cohort-specific missingness thresholds (ALSPAC: >3%; MCS: >20%; BiB: >10%). SNP-level filters were applied to exclude variants with high missingness (>1–5%), low MAF (<0.5–1%), or that violated Hardy-Weinberg equilibrium. Multi-batch datasets were merged and KING [59] was used to remove unexpected relatives, yielding maximal unrelated subsets of genetically inferred European-ancestry children (n≈2,800–8,600 per cohort) and their parents (Supplementary Table 1). Genetic ancestry was inferred using principal component analysis and samples with genetically-inferred European ancestry were identified in each cohort. Finally, each cohort was imputed separately (ALSPAC to the TOPMed r2 panel via the TOPMed server, and MCS/BiB to the HRC panel via the Michigan server) and only well-imputed bi-allelic variants (Minimac R^2^ > 0.8 for ALSPAC/MCS; > 0.9 for BiB; MAF > 1%) were retained (≈5.5–8.1 million SNPs per cohort).

### Calculation of polygenic indices

PGIs were calculated using the most recent, well-powered GWAS summary statistics for conditions on the internalising and externalising spectrum and education-related traits (see Table 1 for an overview). SNP weights were calculated using LDpred2-auto [60], which does not require a validation dataset for tuning of hyper-parameters. LD reference panels were calculated using 1,444,196 HapMap3+ variants [61] and a subset of unrelated European individuals (kinship coefficient < 0.0442) from the target samples, leaving 1,212,346 SNPs for ALSPAC, 1,290,455 SNPs for MCS and 934,933 SNPs for BiB. The --score function in PLINK v1.9 [62] was used to calculate the children’s PGIs as the weighted sum of genotypes across a set of SNPs for each individual. Lastly, to adjust for population stratification, the first ten genetic principal components were regressed out of the PGI within each cohort, and the residualised PGI was used in further analysis.

### Mendelian imputation of parental genotypes

For individuals with genotype data available for only one parent, we used *snipar* [63] with default parameters to impute the missing parental genotypes. Parental PGIs were calculated from the observed and imputed parental genotypes (Minimac4 R^2^ > 0.9) using the pgs.py script provided in the *snipar* package. The Mendelian imputation approach uses inferred identity-by-descent sharing and Mendelian inheritance probabilities to impute missing parental genotypes, and has previously been shown to produce parental genotype estimates with negligible bias [63]. Mendelian imputation increased the number of genotyped trios in our analytic sample (genetically-inferred European ancestry with at least one SDQ measurement) from 1,386 to 5,605 in ALSPAC, 2,083 to 4,555 in MCS, and 178 to 1,032 in BiB. See Supplementary Table 1 for the total number of trios in each cohort post imputation. LDpred2-auto SNP weights (see previous section) were used for parental PGI calculation.

### Exome sequence data preparation

The procedures for generating and quality control of the exome sequencing data are described in detail elsewhere [64]. Briefly, single nucleotide variants (SNVs) and insertions and deletions (indels) were called for samples from ALSPAC (N=11,994), MCS (N=15,050), and BiB (N=11,916) with GATK (version 4.2.4.0 and later releases) following GATK best practices [65]. Sample quality control included the exclusion of suspected sample mismatches, and outlier samples identified using several metrics (e.g., variant counts, heterozygosity). To facilitate variant quality control, a random forest was trained on pre-defined truth sets in each cohort separately. The random forest filtering was then applied with additional genotype-level and missingness filters. Specifically, SNVs were filtered (genotypes were set to missing) using the following thresholds: allele depth (DP) < 5, a heterozygous allele balance ratio (AB) < 0.2, or a genotype quality (GQ) < 20 (ALSPAC) or < 15 (MCS and BiB); Indels were filtered using these thresholds: DP < 10 (ALSPAC and MCS) or < 5 (BiB), AB < 0.3, GQ <10 (ALSPAC) or GQ < 20 (MCS and BiB). Variants that failed the random forest filtering or with missingness > 0.5 after applying the genotype filters were removed. The final dataset included samples for 8,436 children and 3,215 parents in ALSPAC, 7,667 children and 6,925 parents in MCS, and 8,784 children and 2,875 parents in BiB (Supplementary Table 1).

### Rare variant annotation

All variants were annotated using the MANE Select transcript, and loss-of-function confidence (LOFTEE) and deleteriousness scores (CADD) were assigned using the Ensembl Variant Effect Predictor (VEP)[66]. Protein-truncating variants (PTVs) were defined as those classified as “high confidence” by LOFTEE, with a CADD score > 25 (for SNVs), and not located in the last exon or intron. We defined damaging missense variants as those with CADD ≥ 25 and MPC[67] score ≥ 2. We restricted our analysis to rare variants with a cohort-specific internal allele frequency < 0.1% (allele frequency in a subset of unrelated children) and an external allele frequency in gnom-AD [68] V3 of < 3×10^-5^. As BiB has two main ancestry groups, European and South Asian, we restricted our analysis to variants with an internal allele frequency < 0.1% in both genetic ancestry groups.

### Calculation of rare variant burden scores

We calculated a rare variant burden score (RVBS) for each consequence class using the following formula, based on work by Gardener et al. [43]:

$$RVBS_{i,c}=\underset{g\in G}{\sum }I_{g,c}(i)S_{g}$$

where *I_g,c_*(*i*) is an indicator function for whether an individual i has a variant of consequence c in gene g, *S_g_* is the fitness cost for heterozygous carriers of a pLoF allele in gene i estimated in Sun et al. [69], and G is the set of all autosomal genes.

In addition to exome-wide RVBS, we also calculated RVBS for genes in gene sets relevant to neurodevelopment, including gene sets for EA, developmental disorders and autism spectrum disorders (Supplementary Methods).

### Non-response and sampling weights for MCS

MCS employed a nonrandom sampling strategy and sampling weights are provided by the study team to adjust for this [70]. Additionally, we used inverse probability weighting to estimate nonresponse weights to account for the nonrandom missingness of participants at each study sweep. The process of generating the nonresponse weights is described in detail elsewhere [33]. Briefly, we fit a logistic regression to predict whether an individual had complete data for a given association analysis based on a set of predictors associated with attrition including housing tenure, parental years in education, household language, study strata (a composite for country of residence and socioeconomic status), single-parent status, breastfeeding status. We then extracted the predicted probabilities of having complete data for all individuals and used their inverse values as nonresponse weights. We incorporated the product of the UK sampling weight and nonresponse weight in our regression models.

### Testing for associations between genetic measures and traits

First, we tested for an association between a genetic measure and externalising and internalising symptoms in ALSPAC and MCS by estimating a linear mixed-effects model using the *lme4* package in R [71]. This analysis was conducted separately in each cohort, restricting to a subset of unrelated participants with genetically-inferred European ancestry for the common variant analyses. Rare variant association tests were performed in unrelated participants of genetically-inferred European ancestry in ALSPAC, MCS, and BiB, and additionally in unrelated participants of genetically-inferred South Asian ancestry in BiB (See Supplementary Table 1 for sample sizes). For all regression analyses, genetic measures and age-specific SDQ symptom scores were standardized within each cohort to have a mean of 0 and a standard deviation of 1. Models were estimated for all cohort members and in the subset of cohort members with genetic data (observed or imputed) for the full parent-child trio:

$$P_{c,t}=\beta_{G}\times G_{c}+\beta_{age}\times \;age_{c,t}+\beta_{sex}\times \;sex_{c}+u_{c}+\varepsilon_{c,t}$$

where *P_c,t_* is a child *c*’s observed score for externalising or internalising symptoms at assessment age *t; G_c_* is the child’s genetic score; age_*c,t*_ is the child’s age at assessment *t*; sex_*c*_ is the child’s sex; *u_c_* is a per-child random intercept capturing between-individual variation; and *ε_c,t_* is the residual error. All parameters are estimated from the data. The main genetic effect β_G_ can be interpreted as the average genetic effect on the phenotype across the ages. We refer to these models as “population models” and to β_G_ as the “population effect” throughout, to distinguish them from the within-family trio models described below.

To identify outlier associations at specific assessment ages, we also tested for cross-sectional associations at each time point with the following simplified linear model using the *lm* function in R[72]:

$$P_{c}=\beta_{G}\times G_{c}+\beta_{sex}\times sex_{c}+\varepsilon_{c}$$

where *P_c_* is the symptom score at a single age for child *c* regressed on the child’s genetic score and sex, and *ε_c_* is the residual error. The results from these cross-sectional models are shown in Supplementary Figure 1-3.

#### Estimating direct genetic effects

Next, we conducted trio-based analyses to assess whether the association between the symptom score and selected genetic measures reflects direct genetic effects or the influence of non-transmitted parental alleles. We estimated regression models adjusting for the parental genetic measures. In these models, the direct genetic effect, δ, may be estimated by subtracting the effects of non-transmitted alleles, θ_NT_, from transmitted alleles, θ_T,_.[15] These parameters are estimated in the following model:

$$P_{child}\sim \theta_{T}\times G_{T}+\theta_{NT}\times G_{NT}$$

where *G_T_* is the genetic measure for transmitted alleles (the child’s score) and *G_NT_* is the genetic measure for non-transmitted alleles in the parents. This can be further decomposed to:

$$P_{child}=\left(\theta_{T}-\theta_{NT}\right)\times G_{child}+\theta_{NT}\times \left(G_{mother}+G_{father}\right)$$

In this model, the coefficient for the child’s genetic measure, (*θ_T_* − *θ_NT_*), represents direct effects, and the coefficient on the parent’s genetic measures, *θ_NT_* represents the effects of non-transmitted alleles. As the coefficients on parental genetic measures capture not only indirect genetic effects (sometimes called “genetic nurture”) but also the effects of population stratification and parental assortment^15^, we refer to them as “non-transmitted” rather than “indirect” effects.

In practice, we fitted the following model:

$$\begin{array}{c} P_{c,t}=\delta \times G_{c}+\theta_{NT,\;mother}\times G_{mother}+\theta_{NT,\;father}\times G_{father}+\beta_{age}\times \;age_{c,t}\\ +\beta_{sex}\times \;sex_{c}+u_{c}+\varepsilon_{c,t} \end{array}$$

where δ is the direct genetic effect of the child *c*’s alleles, *G_c_. θ_NT_* is the effect estimate of the non-transmitted alleles for the mother and father of child *c*.

We also estimated cross-sectional associations using models that control for parental genetic scores:

$$P_{c}=\delta \times G_{c}+\theta_{NT,\;mother}\times G_{NT,\;mother}+\theta_{NT,\;father}\times G_{NT,\;father}+\beta_{sex}\times sex_{c}$$

where *P_c_* is symptom measured at a single assessment age for child *c* and the covariates are the same as above.

#### Testing for age dynamic genetic effects from childhood to adolescence

To test if age impacted the association between a genetic measure and internalising/externalising symptoms, we fitted an additional model with an age-by-genetic score interaction effect:

$$\begin{array}{c} P_{c,t}=\beta_{G}\times G_{c}+\beta_{age}\times \;age_{c,t}+\gamma \left(G_{c}\times \;age_{c,t}\right)+\beta_{sex}\times sex_{c}\\ +\beta_{sex\;\;\times \;\;age}\times \left(sex_{c}\times \;age_{c,t}\right)+u_{c}+\varepsilon_{c,t} \end{array}$$

where *age_c,t_* is the age of child *c* at assessment t minus the earliest assessment age in each cohort (age 7 in ALSPAC; age 5 in MCS). *β_g_* represents the genetic effect at the earliest assessment age, estimated leveraging data at all ages; γ is the change in the genetic association per unit increase in age; βsex×age is the sex×age interaction term; *u_c_* is the per-child random intercept; and *ε_c,t_* is the residual error.

In trio models, an additional age interaction effect was modeled for the parental genetic values as follows:

$$\begin{array}{c} P_{c}=\delta \times G_{c}+\beta_{age}\times \;age_{c,t}+\gamma \left(G_{c}\times \;age_{c,t}\right)+\theta_{NT,\;mother}\times G_{mother}\\ +\theta_{NT,\;father}\times G_{father}\\ +\phi_{mother}\left(G_{mother}\times \;age_{c,t}\right)+\phi_{father}\left(G_{father}\times \;age_{c,t}\right)+\beta_{sex}\times \;sex_{c}\\ +\beta_{sex\times age}\times \left(sex_{c}\times \;age_{c,t}\right)+u_{c}+\varepsilon_{c,t} \end{array}$$

Where *ϕ_mother_* and *ϕ_father_* represent the change in association with the parental non-transmitted alleles as a function of child’s age, and all other terms are defined as above.

#### Replication of genetic associations in BiB

We estimated population and direct genetic effects of the common and rare variant measures in BiB using linear models. Due to the modest sample size and low proportion of participants with genetic data and at least two SDQ measurements (<20%), the earliest parent-reported SDQ score for each symptom domain was regressed on each genetic measure and relevant covariates; in trio models, parental polygenic scores were also included as predictors.

#### Correction for multiple testing

As we tested for associations between two correlated outcomes, internalising and externalising symptoms, and multiple correlated genetic measures, we applied the Benjamini-Yekutieli (BY) method to adjust for multiple comparisons, maintaining the false discovery rate (FDR) at 5%. For the main analyses, this correction was applied jointly to all p-values for the main effects of all genetic measures (PGIs and RVBS) from three models: 1) population models including all children, 2) population models restricted to children in trios, and 3) trio models estimating direct genetic effects. Effect size estimates from both MCS and ALSPAC were included in this joint correction. The correction for parental non-transmitted allele effects was applied separately. Lastly, BY correction was applied to association estimates from the BiB models following the same procedure used for the main analyses.

### Stepwise Regression

We used a stepwise regression approach to jointly model common and rare genetic measures that explained variation in internalising and externalising symptoms. For each genetic measure with at a least a nominal association with an outcome in the population models, we ranked the variance explained by the genetic measure by comparing the R^2^ for a baseline model - including age and sex as fixed effects and a per-child random effect - to the R^2^ of a model that included the genetic measure. Models were estimated using a subset of probands with both genotype and exome-sequence data in each cohort (N_ALSPAC_=5,424; N_MCS_=4,500). We then added each genetic measure to the baseline linear mixed effects model in a stepwise manner, entering the genetic measure with the greatest marginal R^2^ first. For each additional genetic measure, we compared the log-likelihood of the model including the new genetic measure to the model without it using the log-likelihood ratio test (LLRT). Predictors were retained if the LLRT was significant (p < 0.05/(number of tests per symptom domain)); p-values were adjusted to account for the number of LLRT per symptom domain in each cohort. We assessed the conditional and marginal R^2^ of the final joint model for each outcome in each cohort separately.

## Results

**Figure 1:**
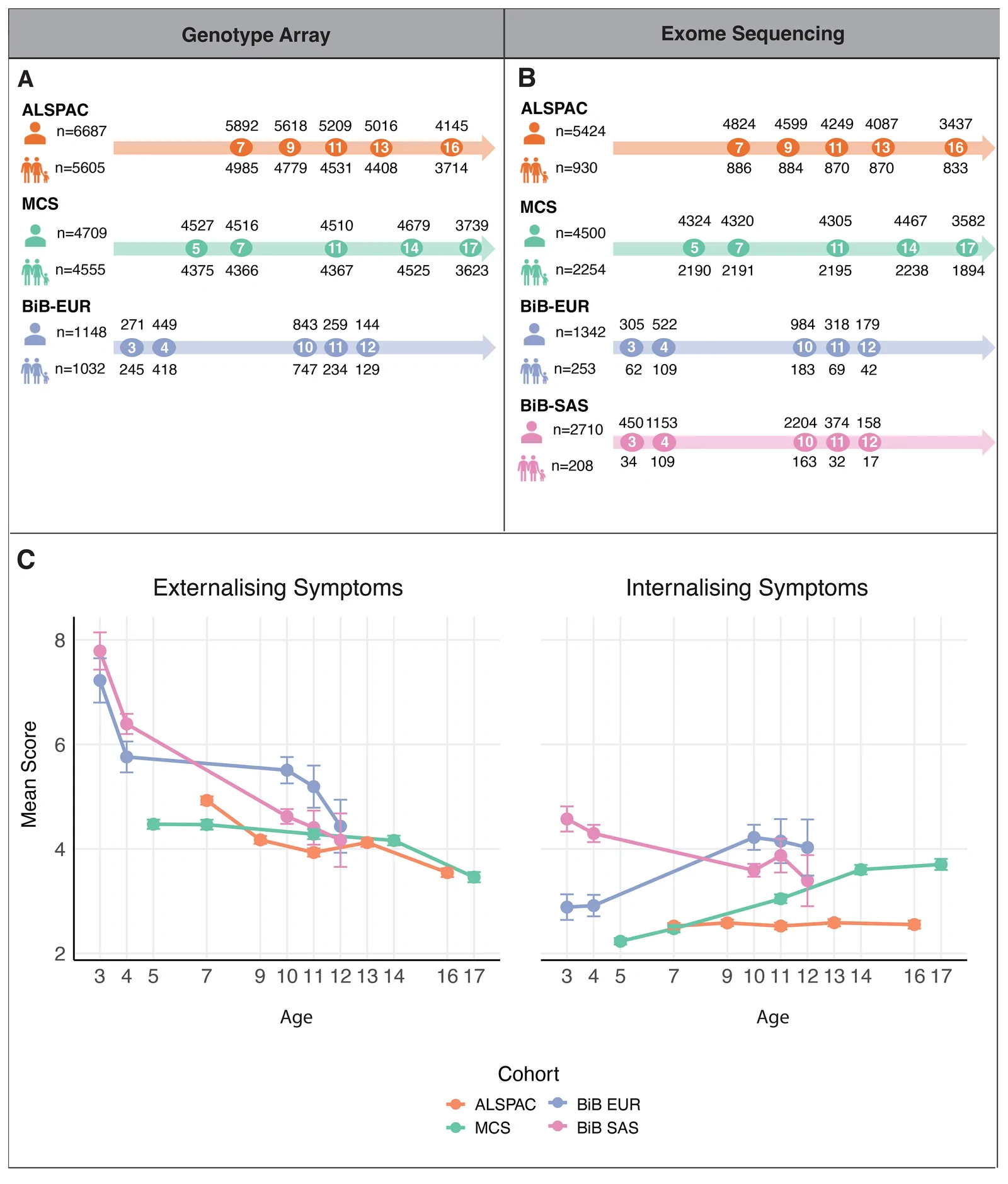
Overview of the birth cohort data. A–B) For each cohort, the number of participants with genotype array (**A**) and exome sequence data (**B**) and at least one SDQ measurement is shown to the left of the arrow. Each assessment age is represented by an opaque circle with the number of children with Strengths and Difficulties Questionnaire (SDQ) and genetic data above the arrows, and the number of children in parent–child trios shown below. Note that, for genotype array data, the latter includes imputed trios following Mendelian imputation of missing parental genotypes for children genotyped as duos. **C**) Mean externalising and internalising SDQ subscale scores at each assessment age for children with genetic data. Error bars represent 95% confidence intervals. EUR: genetically-inferred European ancestry; SAS: genetically-inferred South Asian ancestry.

We analysed genotype array and exome-sequence data from three British birth cohorts that recruited participants across three decades: ALSPAC [47, 48], MCS [50], and BiB [54, 55] (Figure 1A and 1B; Supplementary Table 1). Participants were recruited from different regions of the UK and demographic composition differed between studies (Methods). The sex distribution was comparable across all cohorts, with males comprising 48-51% of the participants. All common variant analyses were conducted in participants of genetically-inferred European ancestry (EUR), while rare variant burden analyses were conducted for each genetically-inferred ancestry group. Only BiB included a second major ancestry group, and analyses were performed separately for participants of genetically-inferred EUR and South Asian ancestry (SAS).

Figure 1C depicts the mean internalising and externalising symptoms score from the SDQ at each assessment stage across the three studies. On average, externalising symptom scores were highest at the earliest assessment age across all studies (Figure 1C; Supplementary Figure 4). Internalising symptom scores remained relatively stable over time, except in MCS, in which scores increased with age. For both symptom domains, mean scores were higher among BiB participants compared to those in MCS and ALSPAC. With the exception of age seven, symptom scores in MCS were higher than in ALSPAC or did not differ significantly. We used data from ALSPAC and MCS for the main analyses because of their larger sample sizes and similarities in the distribution of SDQ symptom scores and the range of assessment ages; BiB analyses are reported separately due to differences in these characteristics (Figure 1; Supplementary Figure 4). In all cohorts, internalising and externalising symptom scores were positively correlated across assessment ages, consistent with partial overlap between these dimensions of childhood mental health [73–75]. However, the modest magnitude of these correlations (r=0.1-0.5) suggests that they capture related but distinct symptom domains (Supplementary Figure 5).

### Influence of common variants on internalising and externalising symptoms across development

**Figure 2:**
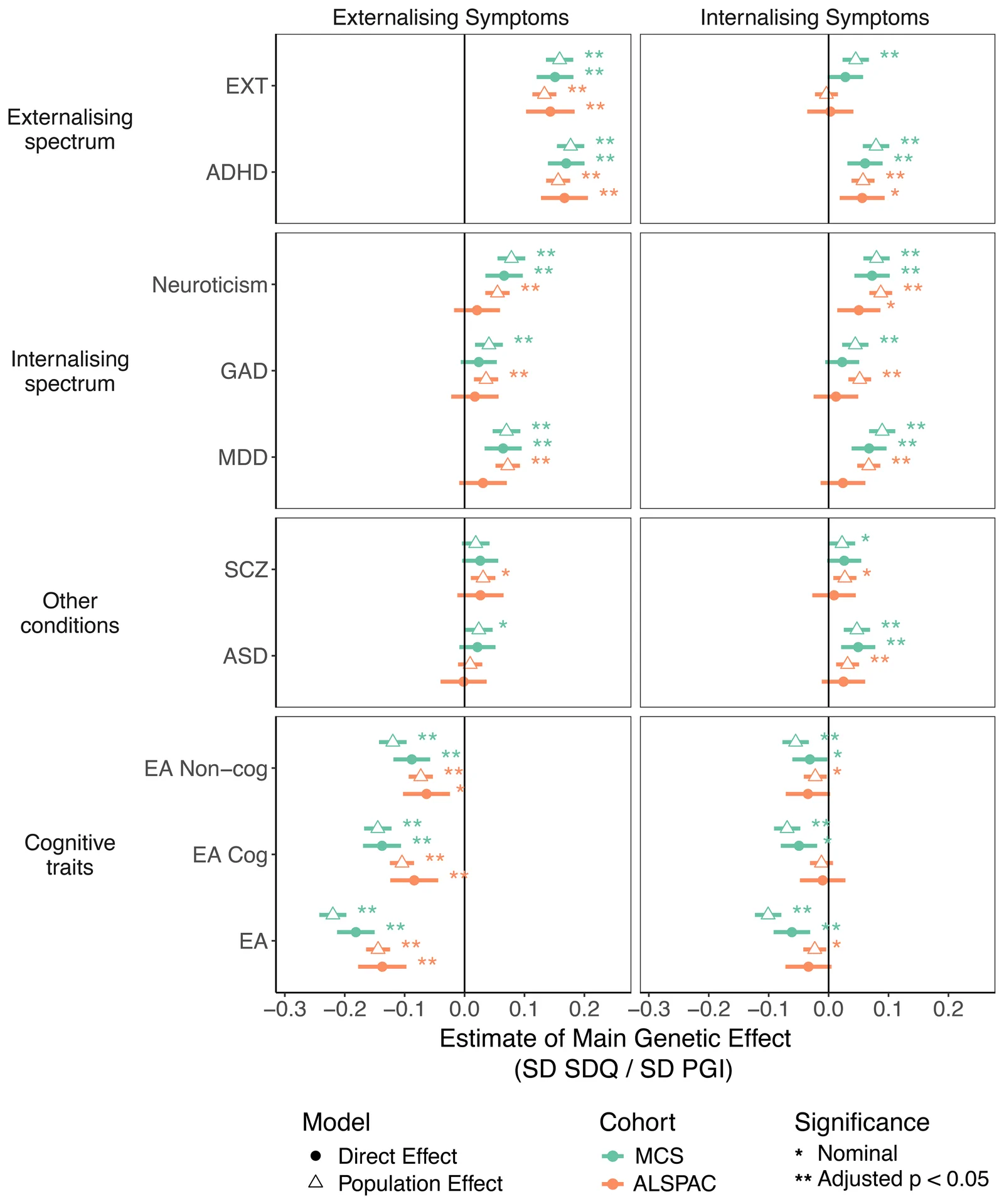
Associations between polygenic indices (PGIs) and childhood and adolescent externalising and internalising symptoms. Standardised effects and 95% confidence intervals estimated for the main effects of the genetic score per standard deviation change in symptom score. Population effect sizes were estimated in the full sample (triangle) and direct genetic effects were estimated in models controlling for parental genetic scores (shaded circles). ADHD: Attention-deficit hyperactivity disorder; ASD: Autism spectrum disorder; EA: educational attainment; EA Cog: cognitive component of EA; EA Non-cog: non-cognitive component of EA; EXT: externalising behaviours; GAD: Generalised anxiety disorder; MDD: Major depressive disorder; SCZ: schizophrenia.

We examined the associations between internalising and externalising symptom scores and PGIs estimated for conditions on the internalising and externalising spectrum, and for education-related traits using linear mixed-effects models. As these traits are known to be phenotypically and genetically correlated [76–78], we first calculated pairwise correlations among PGIs within each cohort and found that most PGIs were significantly correlated, although the correlations were generally weak to moderate in absolute magnitude (r=0-0.6) (Supplementary Figure 6; Supplementary Table 2). Across both ALSPAC and MCS (N_ALSPAC_=6,687; N_MCS_=4,709), higher externalising symptom scores were significantly associated with externalising behaviours and ADHD PGIs (β=0.13 to 0.18, Benjamini-Yekutieli adjusted p(p-adj)<3.49×10^-36^), with smaller but still significant positive associations with the neuroticism and depression PGIs (β=0.06 to 0.08, p-adj<1.1×10^-3^) (Figure 2; Supplementary Table 3). Externalising symptom scores were also significantly associated with lower EA, EA Cog, and EA Non-cog PGIs (β= -0.22 to -0.07, p-adj<2.19×10^-10^). Higher internalising symptom scores were significantly associated with higher ADHD, neuroticism, generalised anxiety disorder, and depression PGIs (β=0.05 to 0.09; p-adj<6.3×10^-3^).

We then sought to determine whether the observed associations between a child’s genotype and their symptoms reflects direct or indirect genetic effects by fitting a “trio model”. As many children only had a single parent genotyped in the study, we used Mendelian imputation to impute the missing parental genotypes. This increased the sample size of genotyped trios with at least one SDQ measurement from 1,386 to 5,605 in ALSPAC and from 2,083 to 4,555 in MCS. Before running the trio model, we confirmed that the results above (i.e., the PGI association with each symptom domain) were consistent whether analysing probands from the full cohort (Population Effect - Whole Cohort) or the subset of probands with at least one parent genotyped (Population Effect - Trios) (Supplementary Figure 7; Supplementary Table 3).

**Figure 3:**
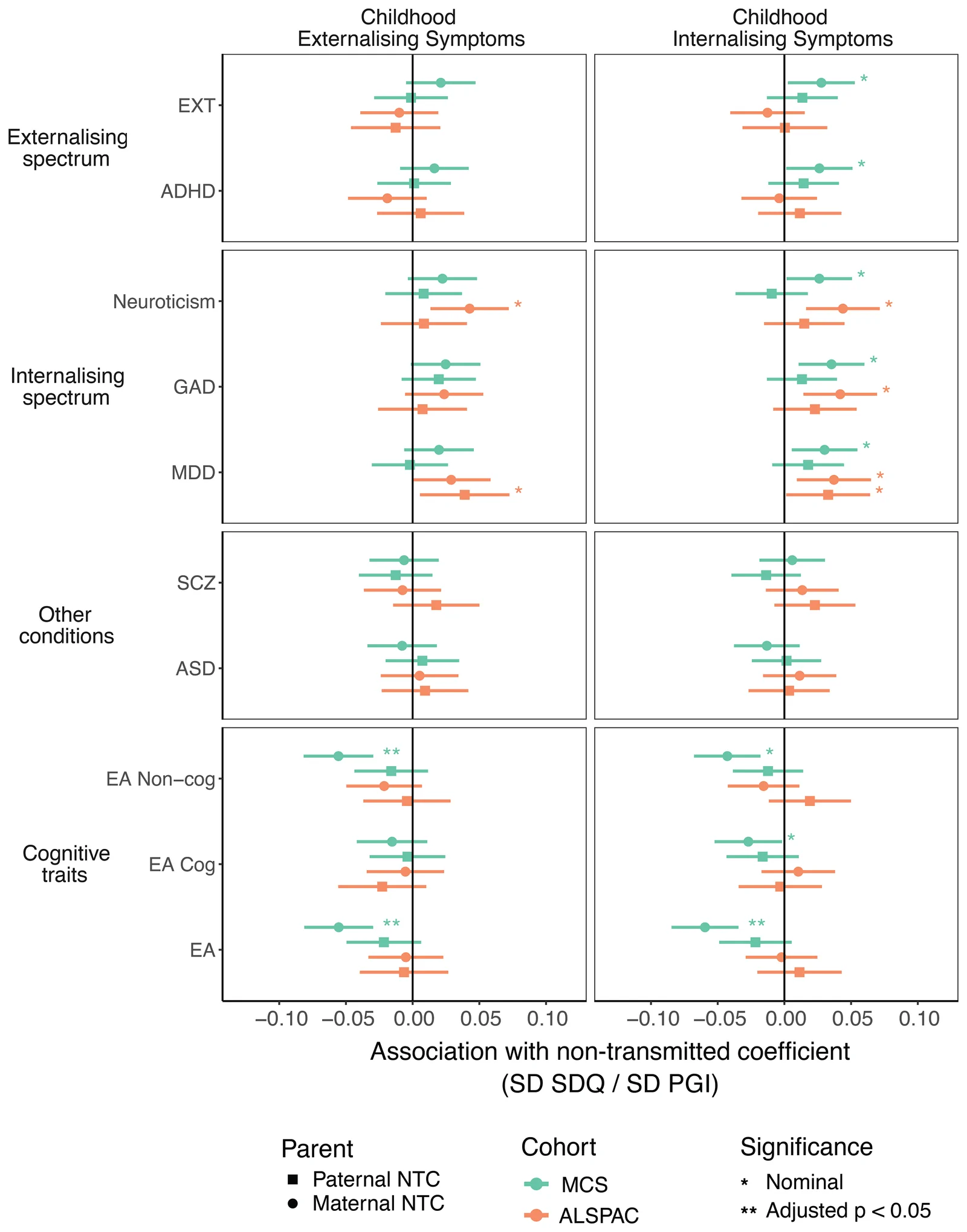
Effect size estimates for parental non-transmitted coefficients (NTCs) for polygenic indices (PGIs) estimated using trio models in MCS and ALSPAC. Error bars represent the 95% confidence interval for the estimate. ADHD: Attention-deficit hyperactivity disorder; ASD: Autism spectrum disorder; EA: educational attainment; EA Cog: cognitive component of EA; EA Non-cog: non-cognitive component of EA; EXT: externalising behaviours; GAD: Generalised anxiety disorder; MDD: Major depressive disorder; SCZ: schizophrenia.

Our findings from the trio model suggest that the associations between externalising symptom scores and PGIs for externalising behaviours and ADHD are explained by direct genetic effects. However, the association with PGIs for EA and EA non-cog may be due to direct genetic effects as well as genetically-influenced maternal traits and behaviours, or potential confounding. The direct effects were modest in magnitude, consistent with the effect sizes in the population models. Specifically, across both cohorts we found direct effects of PGIs for externalising behaviours and ADHD (β=0.14 to 0.17), as well as for education-related traits (β=−0.18 to −0.06) on externalising symptom scores across both cohorts. The only association that did not remain significant (p-adj<0.05) after multiple-testing correction was that for the EA non-cog PGI in ALSPAC (Figure 2; Supplementary Table 3). In contrast, we only saw significant non-transmitted coefficients for the EA and EA non-cog PGIs, and then only for mothers in MCS (β≈-0.06, p-adj<0.04) (Figure 3; Supplementary Table 4). Post-hoc power analyses indicated that we had >80% power at the nominal significance threshold (p<0.05) to detect parental non-transmitted coefficients of β≥0.04 in both cohorts (Supplementary Note 1; Supplementary Figure 8). The null non-transmitted coefficients for the externalising behaviour and ADHD PGIs are therefore unlikely to reflect insufficient statistical power.

For internalising symptoms, we observed positive direct effects for the ADHD and neuroticism PGIs (β=0.05 to 0.07) that were significant in MCS (p-adj<3×10^-3^) and were nominally significant in ALSPAC (p<0.05; p-adj>0.05)(Figure 2; Supplementary Table 3). Among the parental non-transmitted coefficients, only the maternal PGIs for neuroticism, anxiety and depression showed nominally significant associations with internalising symptom scores in both cohorts (β=0.03 to 0.04; p<0.05, p-adj>0.05)(Figure 3; Supplementary Table 4). Several additional significant direct effects were observed in MCS only (p-adj<0.03), including associations of MDD (β=0.07), ASD (β=0.05), and the EA (β=-0.06) PGI with internalising symptom scores and of neuroticism (β=0.07) and MDD (β=0.06) PGIs with externalising symptom scores (Figure 2; Supplementary Table 3).

We observed concordant results for population estimates using SDQ symptom scores measured at a single time point for BiB participants (Methods; Supplementary Figure 9; Supplementary Table 5). For estimates from the trio models (Supplementary Table 4-5), the direction of direct genetic effect was generally consistent with that observed in MCS and ALSPAC, but confidence intervals were wide due to the modest sample size (N=1,037).

Returning to ALSPAC and MCS, we also fitted models with an interaction between age and each PGI to test whether these genetic associations changed across development (Methods). Across externalising and internalising symptoms, only the externalising behaviour PGI showed evidence of an interaction with age in the population models, suggesting that the influence of common variants associated with this trait on externalising symptom scores increased with age (Supplementary Figure 10; Supplementary Table 6). This PGI by age interaction was significant in ALSPAC (β=0.007, p-adj=7.7×10^−10^), and nominally significant in MCS (β=0.002; p<0.05, p-adj>0.05). Moreover, the increasing influence of these variants with age appears to be driven by direct genetic effects: we observed a nominally significant positive interaction between the direct effect of the externalising behaviour PGI and externalising symptom scores in the trio models which was significant in MCS (PGI×age: β=0.006; p-adj=9.5×10^-5^) and nominally significant in ALSPAC (PGI×age: β=0.004; p=0.03, p-adj>0.05) (Supplementary Figure 10; Supplementary Table 6).

### Influence of rare variant burden on internalising and externalising symptoms

**Figure 4:**
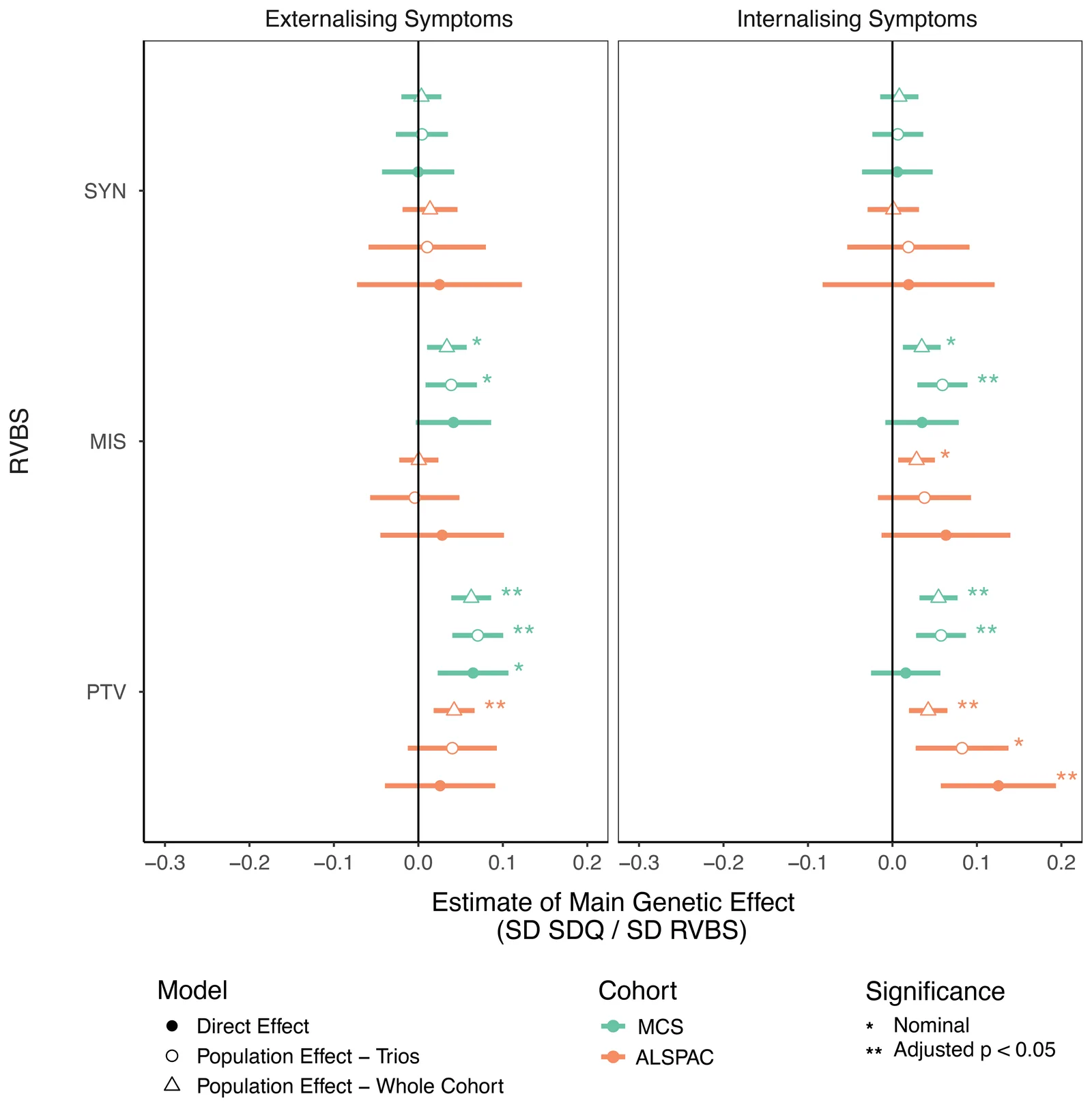
Associations between rare variant burden scores (RVBS) and childhood and adolescent externalising and internalising symptoms. Standardised effects (in standard deviations of SDQ score) and 95% confidence intervals estimated for the main effects of the RVBS per standard deviation change in symptom score for RVBS calculated with three different consequence classes. Population effect sizes were estimated in the full sample (circle) and in a subset of children with parental RVBS (triangle). Shaded circles illustrate direct effect estimates from models controlling for parental genetic scores. MIS: missense; PTV: protein truncating variant; SYN: synonymous.

Next, we tested whether rare genetic variation influences childhood mental health using RVBS, an exome-wide measure of burden calculated separately for three classes of rare variants (protein-truncating, missense, and synonymous) that accounts for genetic evolutionary constraint. In linear mixed effects models considering all children, we observed a significant main effect of RVBS for protein-truncating variants (RVBS_PTV_): higher RVBS_PTV_ was associated with an increase in externalising (β=0.04 to 0.06; p-adj<0.03) and internalising (β=0.04 to 0.06; p-adj<0.01) symptom scores in population models in both cohorts (N_ALSPAC_=5,424; N_MCS_=4,500) (Figure 4; Supplementary Table 7). RVBS for missense variants (RVBS_missense_) showed a nominally significant (p<0.05; p-adj>0.05) association with increased internalising symptom scores in both cohorts (β=0.03 to 0.04), and with increased externalising symptom scores in MCS (β=0.03). Direct effect estimates from trio models were consistent across cohorts for externalising (*Z*=0.97, p=0.33) but not internalising (*Z*=-2.7, p=6.3×10^-3^) symptom scores. RVBS_PTV_ showed a significant direct effect on internalising symptom scores in ALSPAC (β=0.12, p-adj<0.02) but not in MCS (β=0.02, p=0.45). No significant associations were observed for parental non-transmitted coefficients in either cohort (Supplementary Figure 11).

To assess whether the exome-wide associations were driven by specific biological pathways, we re-estimated models using RVBS calculated for gene sets selected for their relevance to neurodevelopment (Supplementary Note 2). No associations survived multiple testing correction in both cohorts, but nominally significant associations consistent in direction with the exome-wide result (β=0.03 to 0.05) were observed for RVBS_PTV_ in genes associated with autism spectrum disorder (the SFARI gene list[79]), developmental disorders (DDG2P[80]), and prioritised from the EA GWAS[81] (Supplementary Figure 12). We additionally estimated RVBS for genes expressed in the central nervous system during prenatal and postnatal development, and genes not expressed in the brain. The results suggest that RVBS associations with symptom scores are primarily explained by variants in genes expressed in the brain, although most associations did not survive correction for multiple testing (Supplementary Note 2, Supplementary Figure 12). This finding was most evident for externalising symptom scores, for which no associations were observed for RVBS for genes not expressed in the brain.

Finally, we estimated associations between RVBS and symptom scores separately in the two main genetically-inferred ancestry groups from BiB: EUR (N=1,342; N_trios_=253) and SAS (N=2,710; N_trios_=208). For the EUR group but not the SAS group, we replicated the positive association between RVBS_PTV_ and both symptom domains in the population models including all children, at a nominal level of significance (β=0.07 to 0.09; p<0.05, p-adj>0.05) (Supplementary Figure 13; Supplementary Table 8). Despite differences in statistical significance, formal tests for heterogeneity did not indicate meaningful differences in effect sizes between the ancestry groups (Bonferroni corrected Z-test p-value > 0.05; Supplementary Table 9). No significant direct effects were observed for either genetic ancestry group, likely due to limited power.

Across all models and cohorts, the association with RVBS for synonymous variants was null, as expected, indicating that the observed associations with PTV and missense RVBS are unlikely to be fully explained by uncontrolled population stratification. Taken together, these results show that burden of deleterious PTVs is associated with both internalising and externalising symptoms in childhood and adolescence. However, effect sizes were small, indicating that these measures of rare genetic variation account for a limited proportion of phenotypic variance.

### Relative contribution of common and rare genetic variation to internalising and externalising symptoms

Our findings so far demonstrate a relationship between childhood mental health and both common and rare genetic variation. To explore this further, we used a stepwise regression approach to assess the relative contribution of each genetic measure to variance in internalising and externalising symptom scores. We started by ranking the genetic measures based on the variance they explained in each symptom domain (marginal *R*^2^) (Supplementary Table 10) and then added them sequentially to a conditional model, retaining a measure only if it significantly improved model fit (log-likelihood ratio test adjusted p-value < 0.05). In the best fitting conditional models, genetic measures explained more variance in externalising symptom scores (4-7%) than internalising symptom scores (~2%) (Supplementary Table 11). Measures of rare genetic variation contributed more to the prediction of internalising symptom scores than externalising symptom scores; specifically, adding them to the baseline model increased the variance explained by the fixed effects by 10-13% for internalising symptom scores and by 2-4% for externalising symptom scores, although the overall variance explained by the rare variant burden scores was low (0.1-0.3%) (Supplementary Table 12).

**Figure 5:**
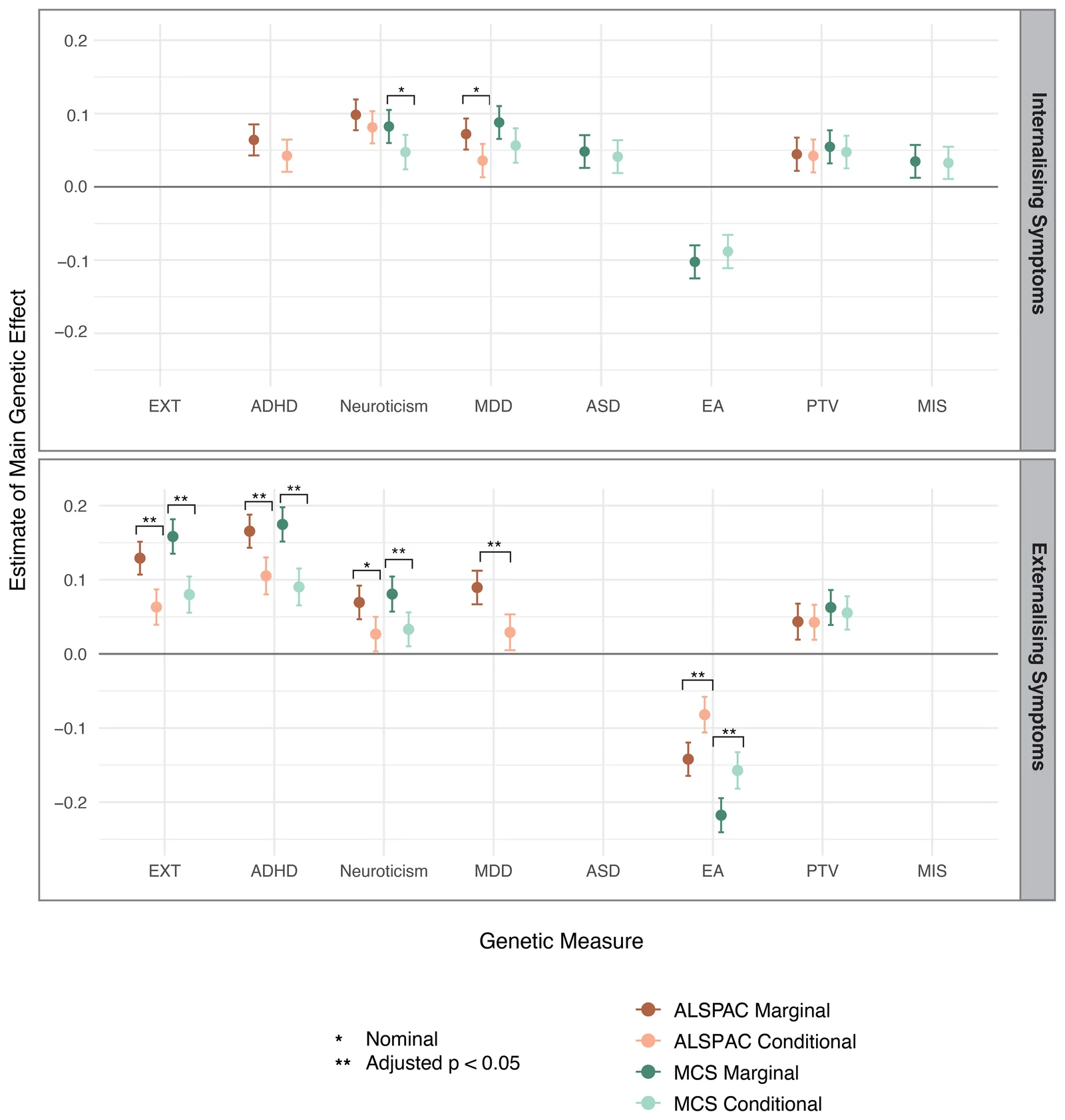
Associations between genetic measures and internalising and externalising symptoms. Standardised effect sizes and 95% confidence intervals are shown for the main effects of each genetic measure, estimated using two models per cohort: (1) marginal models including only the indicated genetic measure and standard covariates, and (2) conditional models including all genetic measures as predictors. Analysis was limited to genetic measures that showed at least a nominally significant association with the symptom domain in the population effect models. Asterisks indicate significant differences between marginal and conditional estimates based on Z-tests. ADHD: Attention-deficit hyperactivity disorder; ASD: Autism spectrum disorder; EA: educational attainment; EXT: externalising behaviours; MIS: missense; MDD: Major depressive disorder; PTV: protein truncating variant.

Next, we assessed whether the associations between genetic measures and symptom scores reflected independent contributions or shared genetic liability across predictors by comparing effect size estimates from conditional and marginal models. Marginal models included a single genetic measure with covariates, while conditional models included multiple genetic measures. We observed an attenuation in the effect sizes of the PGIs in conditional compared to marginal models (Figure 5; Supplementary Table 13), which may reflect a shared genetic liability across these traits, consistent with the concept of a general psychopathology (p) factor[74, 82]. However, for both symptom domains, the effect size estimate for the measures of rare genetic variation remained largely unchanged in the conditional model. These results indicate that the contributions of individual PGIs to externalising and internalising symptoms are not entirely independent when other PGIs are considered. In contrast, the influence of rare genetic variation, indexed by RVBS_PTV_ and RVBS_MIS_, appears to be distinct from that of common genetic factors.

## Discussion

This study provides new evidence for the role of both common and rare genetic variants in shaping internalising and externalising symptoms across childhood and adolescence. Key strengths include: 1) leveraging longitudinal phenotypic data through linear mixed-effects models to account for intra-individual correlations and increase power for genetic association analyses, 2) imputing missing parental genotypes to increase sample size and reduce bias in our common variant analyses, and 3) jointly modelling common and rare genetic variation to address existing gaps in understanding how genetic variation across the allele frequency spectrum influences early-life mental health.

In our analysis of common genetic variation, we found that associations between externalising symptom scores and PGIs for externalising behaviours, ADHD, and the cognitive component of EA were primarily explained by direct genetic effects (Figure 2). These associations were generally consistent across cohorts and correspond with previous work [27–29], though findings have varied across studies [21–23, 25, 26]. We found limited evidence for associations between non-transmitted parental alleles and externalising symptoms despite having adequate power to detect modest effects (Figure 3; Supplementary Note 1). An exception was the negative association between externalising symptom scores and the maternal PGIs for EA and its non-cognitive component in MCS (Figure 3). These results indicate that the significant population effects for these PGIs may also include associations with genetically-influenced maternal characteristics, such as parenting behaviours or the prenatal environment, or unmeasured confounding [15–17]. An association between the maternal non-transmitted coefficient for EA and offspring externalising traits has also been observed in MoBA, a Norwegian birth cohort, although the direction of effect differed from our findings [27, 83]. Our results align more closely with the epidemiological observation that lower household socioeconomic status, including lower parental EA, is associated with increased externalising problems in children [84, 85]. Future studies are needed to clarify the mechanisms underlying intergenerational transmission of risk, including distinguishing true indirect genetic effects from residual confounding. When indirect effects are detected, approaches that account for biases introduced when parental phenotypes are included as mediators in PGI-based models should be considered [86].

For internalising symptoms, we found evidence of a positive direct genetic effect of PGIs for ADHD and neuroticism, reaching at least nominal significance in both cohorts (Figure 2). The nominally significant positive association between internalising symptom scores and maternal PGIs for neuroticism, depression, and anxiety observed in both cohorts may reflect indirect genetic effects or confounding factors, including the possibility that maternal genetic predisposition to these traits influences reporting on their child’s internalising symptoms[87, 88], and warrants further investigation (Figure 3). More broadly, the paucity of associations passing multiple testing correction in the trio models may reflect the lower heritability of internalising traits [36], which would reduce power to detect associations. Further work in larger cohorts, ideally with more sensitive phenotyping of internalising symptoms at younger ages, is also needed to elucidate direct and indirect genetic effects on internalising traits.

One methodological advantage of this study is the use of Mendelian imputation to recover missing parental genotypes, whereas previous studies have typically relied on complete genotyped trios [21, 23, 27, 29]. By using Mendelian imputation, we substantially increased the number of genotyped trios improving power and increasing the significance of several PGI associations. This approach also yielded more consistent findings across cohorts (Supplementary Figures 14-15; Supplementary Note 3) and, in some cases, led to more robust conclusions than analyses restricted to complete trios (Supplementary Figures 16-17). For example, in ALSPAC, the associations between externalising symptom scores and the direct genetic effects of PGIs for externalising behaviours and EA were non-significant when using only observed trios but became significant and increased in magnitude when using imputed trios. Previous research has found evidence of education-related ascertainment bias in these cohorts, showing that children in fully genotyped trios have a higher EA PGI than children who do not have genetic data on both parents [33]. Thus, the changes in our results when using imputed rather than complete genotyped trios likely reflect not only increased sample size but also a reduction in bias introduced by non-random trio ascertainment.

Additionally, the inclusion of rare exonic variation directly addresses a substantial limitation of existing studies, which have predominantly focused on common genetic variants. We demonstrate that a higher burden of deleterious PTVs is associated with increased internalising and externalising symptoms (Figure 4). Notably, we show that RVBS_PTV_ explains additional variance in both symptom domains beyond that accounted for by common genetic variation in conditional models (Figure 5). These findings support the hypothesis that some of the “missing heritability” (i.e., the difference between twin-based and SNP-based heritability estimates) may be attributable to the effects of rare variants, and imply that combining common and rare variant measures could improve prediction of early mental health outcomes. We also sought to refine the rare-variant signal by analysing gene set–specific RVBS_PTV_, and demonstrated several nominally significant consistent associations across the cohorts (Supplementary Figure 12). These findings suggest that deleterious rare variants in genes associated with neurodevelopmental disorders, autism spectrum disorder, and educational attainment are particularly relevant to early-life mental health. However, no associations survived multiple-testing correction, and larger whole-genome–sequenced cohorts will be needed to quantify the total contribution of rare variation (including noncoding variants) and to localise associations to biologically meaningful pathways [89].

Our study has several limitations. First, using SDQ symptom scores from a single rater may have introduced reporter bias, as cross-informant agreement on SDQ is typically low to moderate in population-based samples [90]. Additionally, parental mental health, which is heritable and correlated with offspring genotype, may influence parent-reported SDQ ratings, potentially inducing or inflating associations between offspring genotype and SDQ scores that reflect parental reporting tendencies rather than the child’s symptoms [91–94]. Multi-informant SDQ data were collected across all three cohorts but were either unavailable in our dataset or available only at a limited number of timepoints, precluding their inclusion in the longitudinal analyses. Future work should incorporate multi-informant measures of mental health symptoms [56]. Second, our measures of common and rare genetic variation capture only a portion of the total genetic liability to a trait, which likely contributes to the modest effect sizes observed in the population models. This, in turn, places an upper bound on the magnitude of associations observable in within-family and other downstream analyses. As genetic association study sample sizes grow and methods continue to advance, the genetic contributions to early-life mental health, and the biological pathways through which they act, should be reexamined. Third, our analyses may have been affected by ascertainment bias and non-random missingness across time [48, 95]. To mitigate this, we incorporated weights in MCS and carried out Mendelian imputation of missing parental genotypes in duos with genotype array data. The generally consistent common variant associations observed across cohorts suggest that this mitigation was successful. However, we did not impute missing parental genotypes for the exome sequence data since parents were typically only sequenced if both were available. This reduced our power to detect direct effects of rare variants (Supplementary Note 1), and our results may have been influenced by the non-random ascertainment of trios.

In summary, our findings provide new insights into the genetic architecture of early-life mental health and suggest several promising directions for future research. As larger and more diverse cohorts of sequenced trios become available, it will be important to examine the contribution of genetic variation across the allele frequency spectrum to internalising and externalising problems across development in varied contexts and genetic ancestry groups. Our results also demonstrate that common and rare variants jointly contribute to early-life mental health, highlighting the need to clarify the interplay between these types of genetic variation.

## Supplementary Material

Supplementary Information

Supplementary Tables

## Figures and Tables

**Table 1:** Genome-wide association studies used to derive the polygenic indices (PGIs) included in the common variant analyses. The table reports the discovery phenotype, sample size, SNP-based heritability (h^2^_SNP_), and reference for each PGI. For binary traits, sample size is given as the number of cases and controls. For the EA, externalising behaviours, and neuroticism PGIs, 23andMe participants were excluded from the summary statistics used for PGI estimation due to data-use restrictions.

Trait	Abbrevia tion	Discovery Phenotype/GWAS	Sample Size	h^2^_SNP_	Reference
Educational attainment	EA	Years of schooling completed.	765,283	0.136 (0.004)	Okbay et al., 2022[96]
Cognitive component of educational attainment	EA Cog	Latent genetic factor shared between educational attainment and cognitive performance estimated using GWAS-by-subtraction.	257,700	0.192 (0.006)	Demange et al., 2021[77]
Non-cognitive component of educational attainment	EA Non-cog	Latent genetic component of educational attainment not shared with cognitive performance estimated using GWAS-by-subtraction.	510,795	0.064 (0.002)	Demange et al., 2021[77]
Autism	ASD	Meta-analysis of Danish iPSYCH ASD sample and five Psychiatric Genomics Consortium cohorts	18,381 cases; 27,969 controls	0.118 (0.01)	Grove et al., 2019[97]
Schizophrenia	SCZ	Meta-analysis of Psychiatric Genomics Consortium cohorts. Restricted to genetically-inferred European ancestry samples.	53,386 cases; 77,528 controls	0.24 (0.007)	Trubetskoy et al., 2022[98]
Major depressive disorder	MDD	Meta-analysis of two GWAS of lifetime depressive disorders	148,776 cases; 357,585 controls	0.087 (0.004)	Wray et al., 2018[99]; Mitchell et al., 2022[100]
Generalised anxiety disorder	GAD	GWAS of lifetime anxiety disorder in UK Biobank	25,453 cases; 58,113 controls	0.26 (0.01)	Purves et al., 2020[101]
Externalising behaviours	EXT	Latent externalising factor from multivariate GWAS of seven externalising traits	1,045,957	0.063 (0.003)	Karlsson Linner et al., 2021[102]
Attention deficit hyperactivity disorder	ADHD	Meta-analysis of Danish iPSYCH, deCODE and Psychiatric Genomics Consortium cohorts	38,691 cases; 186,843 controls	0.14 (0.01)	Demontis et al., 2023[103]
Neuroticism		Meta-analysis of UK Biobank, 23andMe and Genetics of Personality Consortium	449,484	0.100 (0.003)	Nagel et al., 2018[104]

## Data Availability

Researchers can apply to access data from ALSPAC (https://www.bristol.ac.uk/alspac/researchers/access/), MCS (https://cls.ucl.ac.uk/dataaccess-training/data-access/), and BiB (https://bib.vergelabs.dev/research/how-to-access-data/). The ALSPAC study website contains details of all the data that is available through a fully searchable data dictionary and variable search tool: http://www.bristol.ac.uk/alspac/researchers/our-data/.

## Code Availability

Code used for this analysis is available upon request from the corresponding author.
