# Supplementary material for: The Contribution of Common and Rare Genetic Variation to Emotional and Behavioural Symptoms in Childhood and Adolescence
Source: Mol Psychiatry. Author manuscript; Available in PMC 2026 Oct 11. (PMC7619542; doi:10.1038/s41380-026-03851-6)
Supplement: Supplementary file 1 [file EMS218325-supplement-Supplementary_Information.pdf]

Supplementary Methods

Study design and participants

Avon Longitudinal Study of Parents and Children (ALSPAC)

Pregnant women resident in Avon, UK, with expected dates of delivery between 1st April 1991 and 31st December 1992 were invited to take part in the study. 20,248 pregnancies were identified as being eligible and the initial number of pregnancies enrolled was 14,541 [1–3]. Of the initial pregnancies, there was a total of 14,676 foetuses, resulting in 14,062 live births and 13,988 children who were alive at 1 year of age. When the oldest children were approximately 7 years of age, the initial sample was increased by enrolling children who did not join the study originally. The number of new pregnancies not in the initial sample (Phase I enrolment) that are currently represented in the released data and reflecting enrolment status at the age of 24 is 906, resulting in an additional 913 children being enrolled (456, 262 and 195 recruited during Phases II, III and IV respectively). The total sample size for analyses using any data collected after the age of seven is therefore 15,447 pregnancies, resulting in 15,658 foetuses. Of these, 14,901 children were alive at 1 year of age.

Of the original 14,541 pregnancies, 338 were from a woman who had already enrolled with a previous pregnancy, meaning 14,203 unique mothers were initially enrolled in the study. As a result of the additional phases of recruitment, a further 630 women who did not enrol originally have provided data since their child was 7 years of age. This provides a total of 14,833 unique women (G0 mothers) enrolled in ALSPAC as of September 2021.

G0 partners were invited to complete questionnaires by the mothers at the start of the study and they were not formally enrolled at that time. 12,113 G0 partners have been in contact with the study by providing data and/or formally enrolling when this started in 2010. 3,807 G0 partners are currently enrolled.

Preparation of genotype data

ALSPAC

Processing of the genotype array data by the ALSPAC study team was performed in two batches[1] (https://proposals.epi.bristol.ac.uk/alspac_omics_data_catalogue.html#orgc66ee19). Batch one included 8,884 mothers genotyped using the Illumina Human 660W chip and 8,932 children genotyped using the HumanHap550 quad chip. The ALSPAC study team removed samples with missingness rate < 3%, heterozygosity outliers, and mismatched sex as well as SNPs with missingness rate > 5%, MAF < 1%, and HWE test p-value > $1x10^{-7}$. SNPs with missingness rate >1% across all samples in the merged batch were also removed.

Another 2,189 parents (mothers and partners) were genotyped on the CoreExome array chip in the second batch[4]. We applied the following additional quality control filters to the batch two array data: removal of autosomal SNPs with MAF <0.5%, missingness rate >3%, and

that failed the HWE test (p-value < $1x10^{-5}$). An additional seven samples with missingness >3% were excluded.

We merged the two batches and used KING[5] for relatedness inference. We excluded 152 samples who had unexpected first-degree relationships but were reported to be from different families. An additional 16 samples that did not match available exome sequencing data supposedly for the same individuals were removed. This left array data for 8,831 children, 9,302 mothers, and 1,706 fathers for further analysis. We identified a maximal subset of unrelated children (N=8,637) using an iterative approach: children with the highest number of genetically inferred relatives (third degree of closer) were iteratively removed until each remaining child had no inferred relatives.

To identify individuals with genetically inferred European ancestry, principal component analysis (PCA) was first performed in the maximal subset of unrelated children and ALSPAC samples were projected onto samples from 1,000 Genomes phase 3 individuals[6] using the smartpca function from EIGENSOFT version 7.2.1[7]. For this analysis, we used 90,563 LD-pruned SNPs (pairwise r2 <0.2 in batches of 50 SNPs with sliding windows of 5) with MAF > 5% after removal of 24 regions with high or long-range LD, including the HLA[8]. All unrelated ALSPAC samples projected onto European ancestry samples. We then projected all remaining ALSPAC samples into this principal component space and calculated loadings for the first ten principal components.

Each batch of genotype array data was imputed separately to the TOPMed r2 reference panel using the TOPMed Imputation Server[9–11]. Variants that had Minimac4 R^2 > 0.8 and MAF > 1% in both batches were retained, leaving 8,069,962 variants for downstream analysis.

MCS

Collection and processing of the MCS genotype array data is described in detail elsewhere[12]. Briefly, MCS participants were genotyped using the Infinium Global Screening Array-24 v1.0. Samples with missingness rate > 20%, high or low heterozygosity (+/- 5 SDs), and sex mismatches were removed. Autosomal SNPs with missing rate <5%, MAF >0.5%, and that passed the HWE test (p-value > $1x10^{-5}$) in a subset of unrelated individuals with genetically inferred European ancestry were retained. An additional 283 samples with missingness rate >5% were removed.

A set of unrelated individuals of genetically inferred European ancestry was identified in MCS following procedures similar to those applied in ALSPAC (as described in Huang *et al. [13]*). Individuals with unexpected familial relationships were removed, leaving 6,153 children, 6,646 mothers and 3,835 fathers [13].

MCS data was imputed to the Haplotype Reference Consortium[11] (Version r1.1 2016) reference panel using the Michigan Imputation Server[10]. After imputation, multi-allelic SNPs and SNPs with Minimac4 R^2 < 0.8 and MAF < 1% were removed, leaving 7,404,321 well-imputed SNPs.

BiB

BiB samples were genotyped using two chips, the Infinium CoreExome-24 v.1.1 BeadChip (~550 K SNPs), and (2) the Infinium GSA-24 v.1 (~640 K SNPs). Samples with missingness >10% and sex mismatches were removed. Autosomal SNPs with missing rate <5%, MAF >0.5% and with a HWE test p-value < $1x10^{-5}$ in a subset of genetically-inferred EUR ancestry individuals were excluded.

The two batches were merged and KING was used for relatedness inference. We excluded 465 samples with unexpected familial relationships. Genetic ancestry inference was conducted following the same procedures as in ALSPAC and MCS [14]. Within the subset of genetically-inferred European ancestry children (N=3,019), a maximal set of unrelated children was identified (N=2,832), as described above.

Each batch of genotype array data was imputed to the Haplotype Reference Consortium[11] (Version r1.1 2016) reference panel using the Michigan Imputation Server[10] separately. After imputation, multi-allelic SNPs and SNPs with Minimac4 R^2 < 0.9 and MAF < 1% were removed. After merging the batches, 5,462,803 variants remained.

Testing for associations with gene set rare variant burden scores

We used linear mixed effects models (Methods) to test associations between deleterious rare variants in predefined gene sets and internalising and externalising symptoms (marginal models). For each gene set, we calculated the rare variant burden score (RVBS) (Methods) by restricting to protein truncating variants (PTV) located in genes within the set. As a negative control, we estimated the gene set RVBS for synonymous variants. Gene sets were selected for their relevance to neurodevelopment and brain function and included:

- Educational attainment (N=1,838): Genes prioritised using DEPICT (FDR<5%) in the educational attainment GWAS from Lee et al. [15].
- Developmental disorders: autosomal recessive (N=635) and autosomal dominant (N=336) genes curated in DDG2P[16]. Most of these are associated with disorders that affect the brain. (https://www.deciphergenomics.org/ddd/ddgenes).
- Autism spectrum disorder (N=1,176): all genes catalogued in the SFARI Gene database (all score categories) [17].
- Brain expression: genes assigned to co-expressed modules enriched for expression in prenatal brain (N=6,808), postnatal brain (N=6,078), or undetected in brain (N=3,028), based on Li et al [18].

All gene sets were intersected with 19,230 autosomal protein-coding genes from Ensembl. Supplementary Figure 15 shows the number of overlapping genes across the educational attainment, developmental disorder, and autism spectrum disorder gene sets.

Next, we estimated a joint (conditional) model including the educational attainment, developmental disorder, and autism spectrum disorder gene set-specific RVBS in the same linear mixed effect model. Conditional models were estimated for all children with exome sequence data and SDQ scores (population effect - whole cohort). We used Z-tests to compare effect size estimates from the marginal and joint models for each gene set-specific

RVBS. The brain-expression gene sets were analysed in marginal models only and were not included in the joint conditional model.

Comparing main genetic effect estimates between cohorts before and after Mendelian imputation

To compare estimates of the main PGI effects between cohorts before and after Mendelian imputation of missing parental genotypes, we estimated weighted Deming regression models. For each symptom domain, fixed-effect estimates and their standard errors for each genetic measure were extracted from two sets of models: 1) *population effects in trios* and 2) *direct genetic effects* from full trio models. A Deming regression model with the following equation was estimated:

$$\hat{\beta}_{ALSPAC} = \alpha + \beta\hat{\beta}_{MCS} + \varepsilon$$

with observation-specific standard errors supplied as weights for both axes.

- Slope (β) quantifies proportional bias: how closely a one-unit change in MCS corresponds to a one-unit change in ALSPAC.
- Intercept (α) quantifies constant bias: whether effect sizes in one cohort are shifted by a fixed amount relative to the other.

Lin's concordance correlation coefficient (CCC) was also computed as a single summary metric that jointly quantifies both constant and proportional bias. Lin's CCC ranges from –1 (perfect discordance) to +1 (perfect concordance).

Post-hoc power analysis for trio models

We conducted a post-hoc simulation-based power analysis using the simr package in R[19] to estimate the statistical power our study had to detect direct genetic effects, maternal non-transmitted coefficients (NTCs), and paternal NTCs in our trio models. Power was estimated separately for each cohort (ALSPAC, MCS) and each genetic measure type (PGI, RVBS), and across a range of effect sizes (PGI direct effects: β=0.01 to 0.20; PGI NTCs and RVBS NTCs: β=0.01 to 0.10; RVBS direct effects: β=0.01 to 0.15). For each combination of cohort, genetic measure type, predictor (direct effect or NTC), and effect size, we conducted 1,000 simulations and recorded the proportion in which the association with the predictor reached nominal significance ($p<0.05$).

Variance components and covariate effects were extracted from fitted trio models in our analytical sample, including the random intercept variance, the residual variance, and the fixed-effect estimates for age and sex. The number of timepoints per child was sampled from the empirical distribution of timepoints contributing to each trio analysis to capture patterns of attrition and missingness over time. Child, maternal, and paternal genetic measures were jointly drawn from a multivariate normal distribution using a theoretical covariance matrix in which each genetic measure had unit variance, the parent-offspring covariance was set to 0.55 (the expected Mendelian transmission correlation of 0.5 plus a small upward correction reflecting moderate assortative mating), and the between-parent covariance was set to 0.15

to reflect moderate assortative mating, consistent with published estimates for education- and mental health-related traits [20, 21]. The outcome was simulated as a linear combination of age and sex fixed effects, the target genetic effect, a per-child random intercept, and residual error.

Supplementary Figures

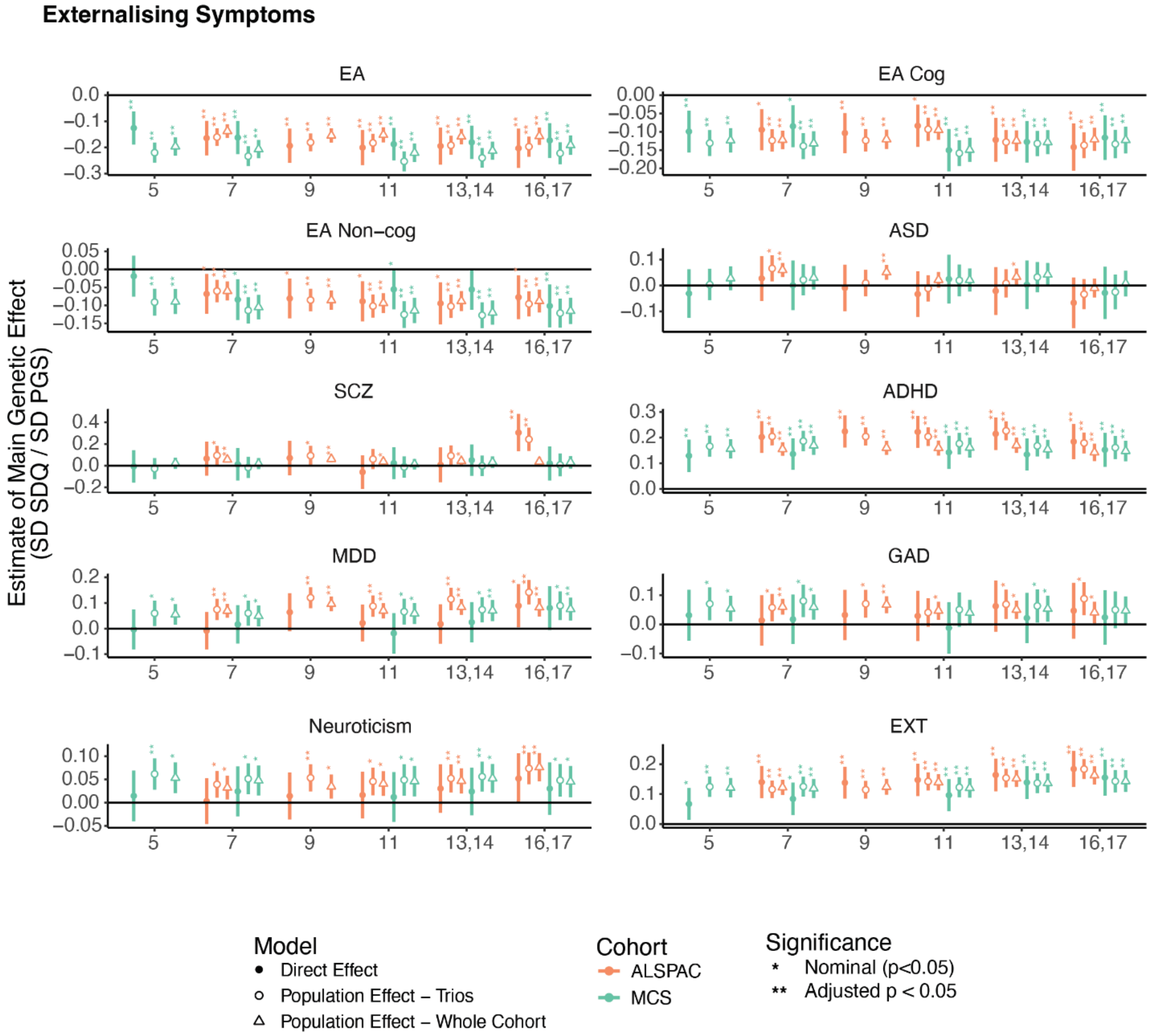

Supplementary Figure 1. Cross-sectional associations between polygenic indices (PGIs) and childhood and adolescent externalising symptoms. Effect sizes represent the standard deviation change in SDQ symptom score per standard deviation increase in the PGI, with 95% confidence intervals. Population effect sizes were estimated in the full sample (circle) and in a subset of children with parental PGIs (triangle). Shaded circles illustrate direct effect estimates from models controlling for parental genetic scores. ADHD: Attention-deficit hyperactivity disorder; ASD: Autism spectrum disorder; EA: educational attainment; EA Cog: cognitive component of EA; EA Non-cog: non-cognitive component of EA; EXT: externalising behaviours; GAD: Generalised anxiety disorder; MDD: Major depressive disorder; SCZ: schizophrenia.

Internalising Symptoms

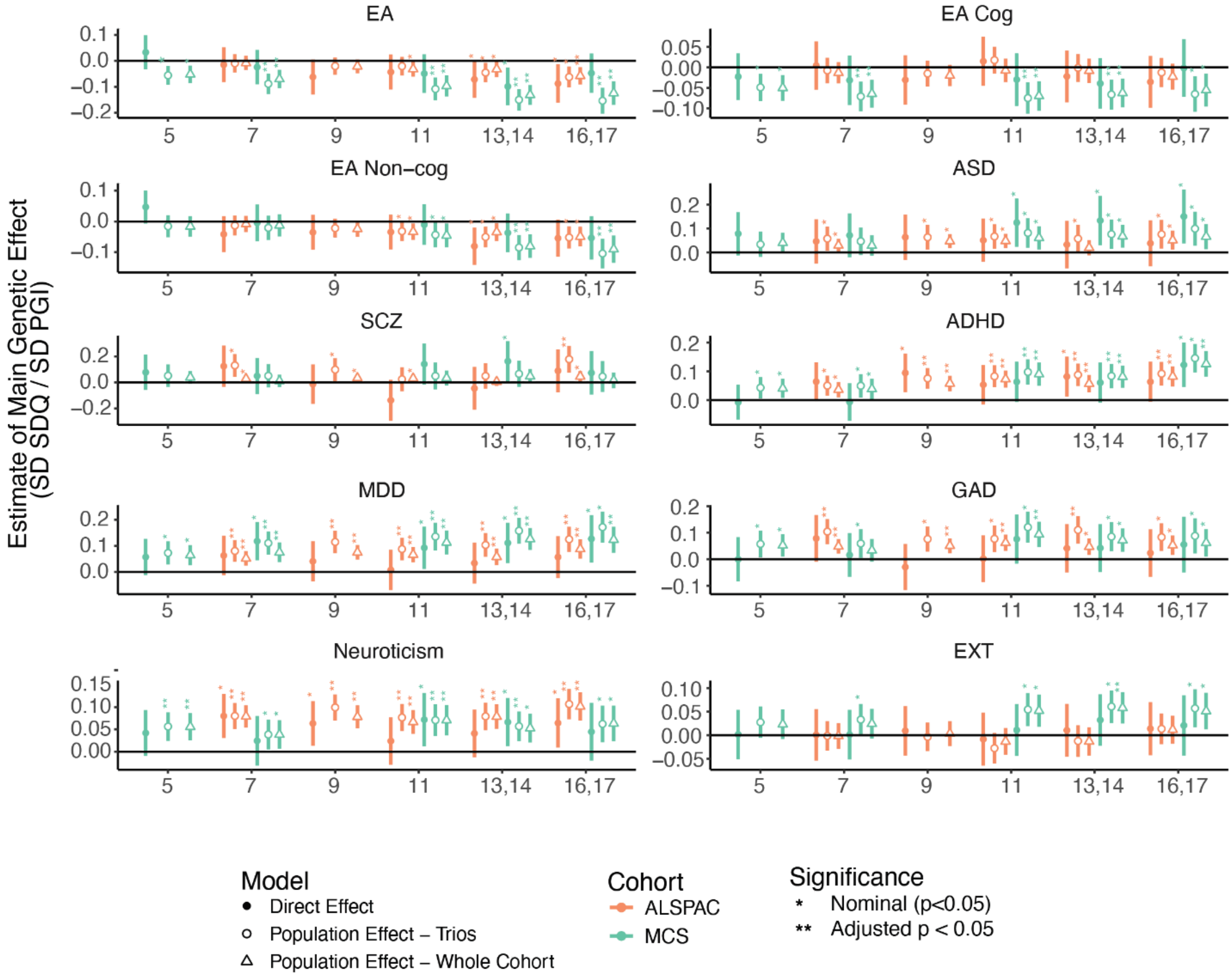

Supplementary Figure 2. Cross-sectional associations between polygenic indices (PGIs) and childhood and adolescent internalising symptoms. Effect sizes represent the standard deviation change in SDQ symptom score per standard deviation increase in the PGI, with 95% confidence intervals.. Population effect sizes were estimated in the full sample (circle) and in a subset of children with parental PGIs (triangle). Shaded circles illustrate direct effect estimates from models controlling for parental genetic scores. ADHD: Attention-deficit hyperactivity disorder; ASD: Autism spectrum disorder; EA: educational attainment; EA Cog: cognitive component of EA; EA Non-cog: non-cognitive component of EA; EXT: externalising behaviours; GAD: Generalised anxiety disorder; MDD: Major depressive disorder; SCZ: schizophrenia.

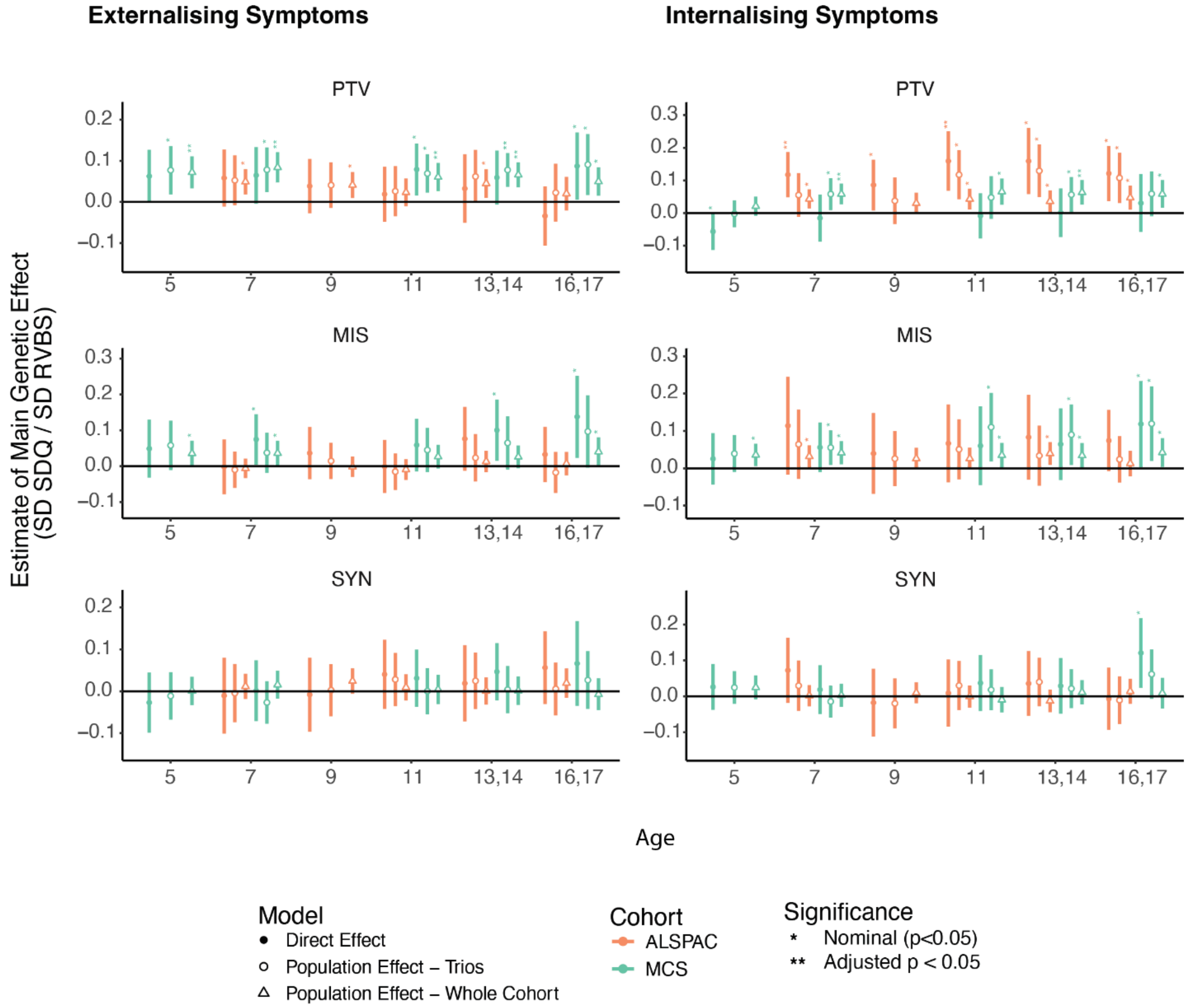

Supplementary Figure 3. Cross-sectional associations between rare variant burden scores (RVBS) and childhood and adolescent extermalising and internalising symptoms. Effect sizes represent the standard deviation change in SDQ symptom score per standard deviation increase in the RVBS, with 95% confidence intervals.. Population effect sizes were estimated in the full sample (circle) and in a subset of children with parental RVBS (triangle). Shaded circles illustrate direct effect estimates from models controlling for parental genetic scores. MIS: missense; PTV: protein truncating variant; SYN: synonymous.

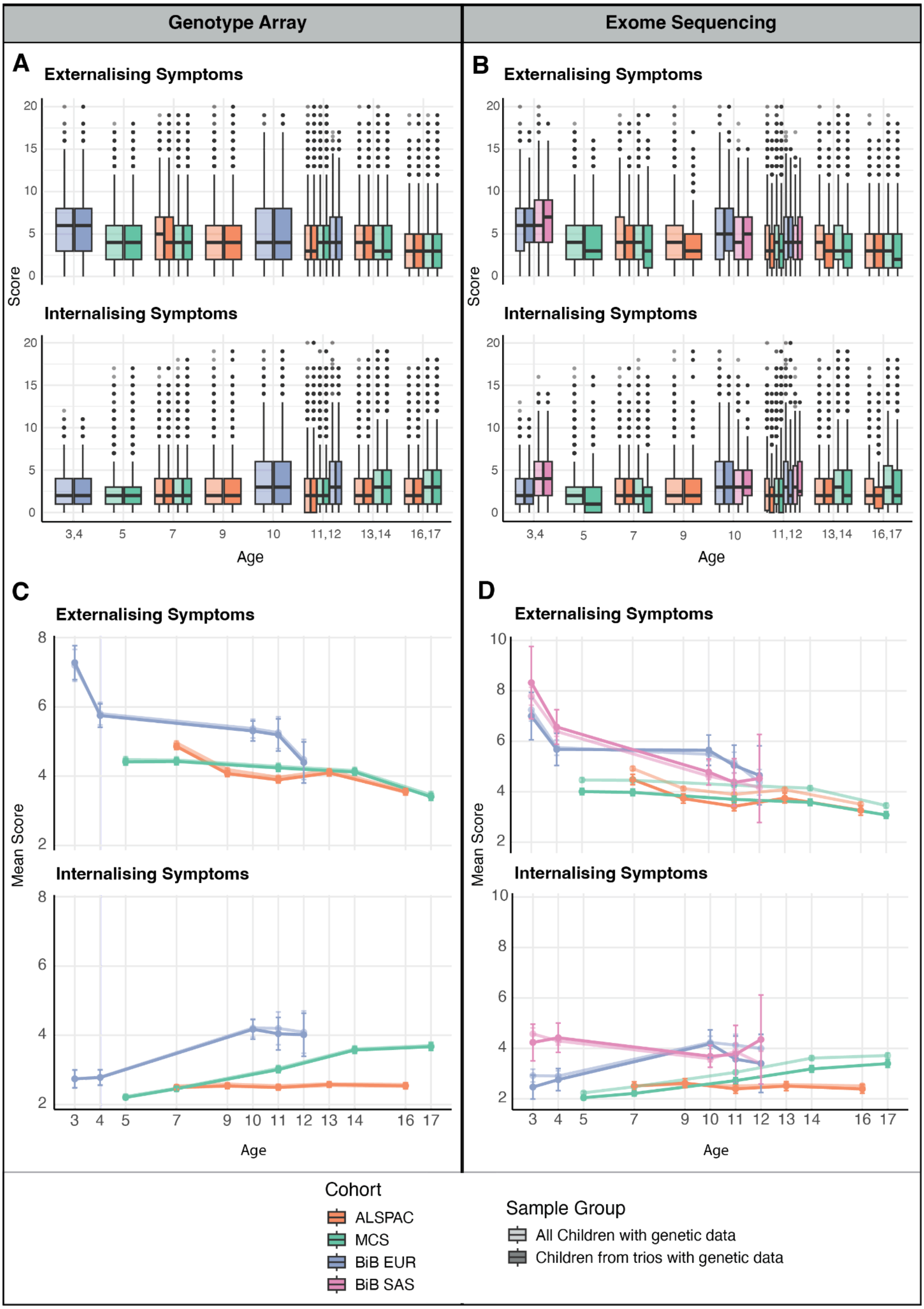

Supplementary Figure 4. Overview of externalising and internalising symptom scores across time for each cohort. A-B) Box plots showing externalising and internalising SDQ subscale scores at each assessment age for children with genotype array and exome sequencing data in each cohort (See Figure 1 for sample sizes). Outliers are defined as values more than 1.5 times the interquartile

range above the third quartile or below the first quartile. C-D) Mean externalising and internalising SDQ subscale scores at each assessment age for children with genotype array and exome sequencing data in each cohort . Error bars represent 95% confidence intervals. Translucent boxes/lines represent all children with genetic data and opaque boxes/lines represent children from trios with genetic data (including those from imputed trios following Mendelian imputation of genotype array data). ALSPAC: the Avon Longitudinal Study of Parents and Children; BIB: the Born in Bradford study; EUR: genetically-determined European ancestry; MCS: Millenium Cohort Study; SAS: genetically-determined South Asian ancestry.

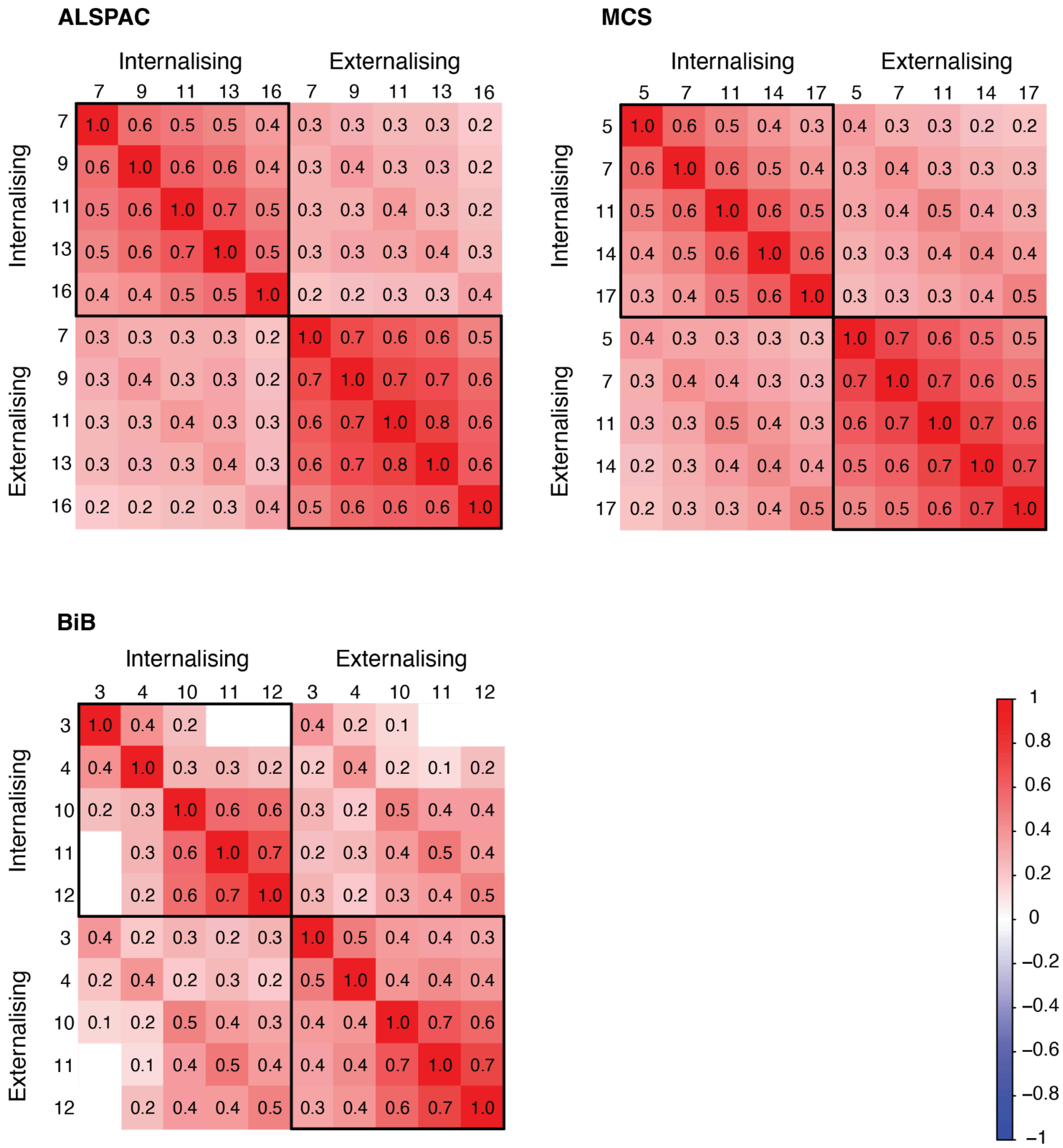

Supplementary Figure 5. Correlations between internalising and externalising symptoms across assessment ages in ALSPAC, MCS, and BiB. Heatmap shows Pearson correlation coefficients estimated between internalising and externalising symptom scores at each available assessment age within each cohort. Warmer colours indicate stronger positive correlations. Only correlations that passed Bonferroni correction for multiple testing ($p < 0.05/135$, corresponding to 45 tests in 3 cohorts) are shown. ALSPAC: Avon Longitudinal Study of Parents and Children; MCS: Millennium Cohort Study; BiB: Born in Bradford.

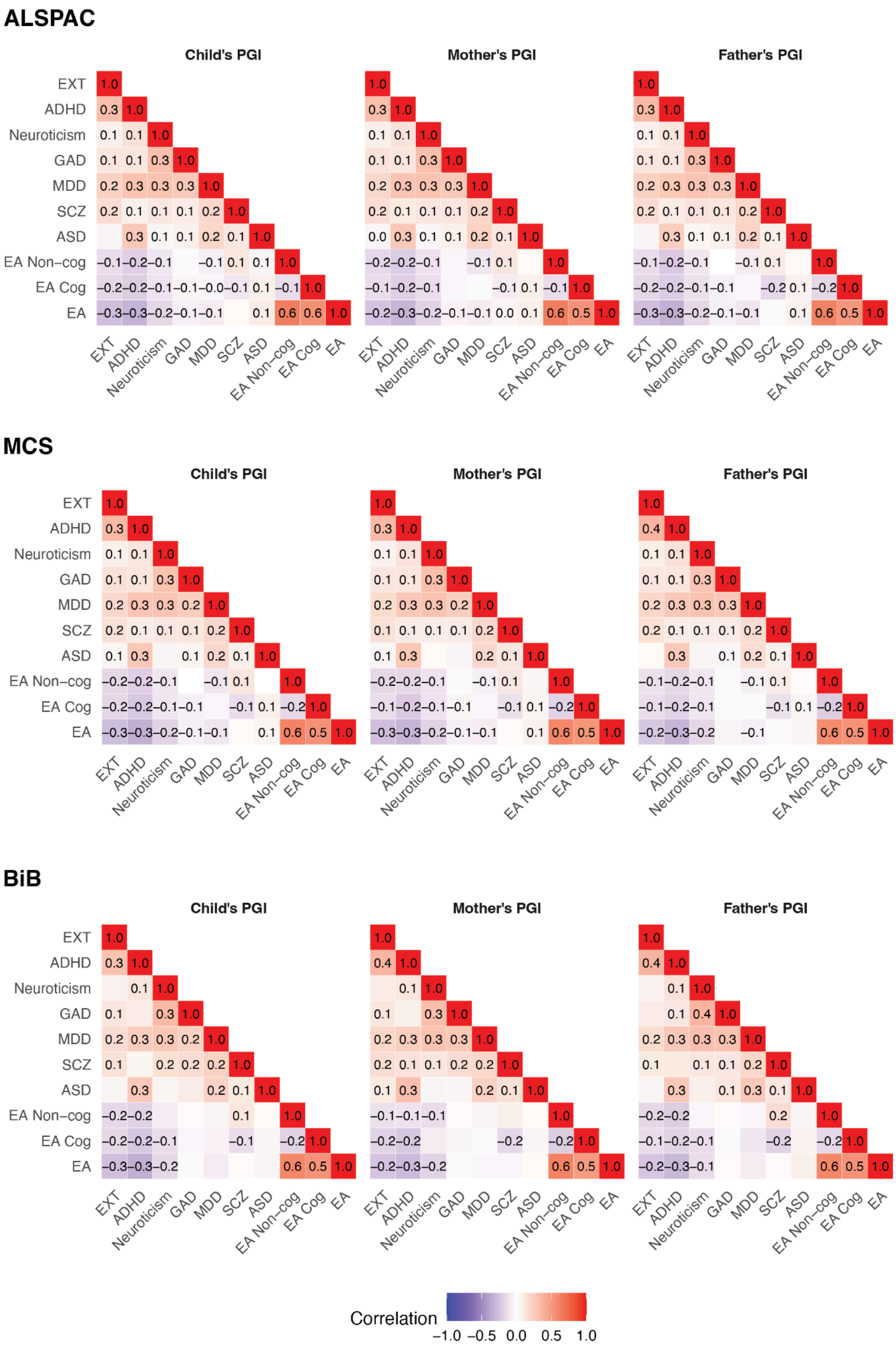

Supplementary Figure 6. Correlations between polygenic indices (PGIs) in ALSPAC, MCS, and BiB. Heatmap shows Pearson correlation coefficients for pairwise associations among the PGIs within each cohort. Correlations were estimated separately within each cohort using all available participants included in the linear mixed effects models for common variant associations. Warmer colours indicate stronger positive correlations. Only correlations that passed Bonferroni correction for

multiple testing ($p < 0.05/135$, corresponding to 45 tests in 3 cohorts) are shown. ALSPAC: Avon Longitudinal Study of Parents and Children; ADHD: Attention-deficit hyperactivity disorder; ASD: Autism spectrum disorder; BiB-EUR: Geneitcally-inferred European ancestry participants from Born in Bradford; EA: educational attainment; EA Cog: cognitive component of EA; EA Non-cog: non-cognitive component of EA; EXT: externalising behaviours; GAD: Generalised anxiety disorder; MCS: Millennium Cohort Study; MDD: Major depressive disorder; SCZ: schizophrenia.

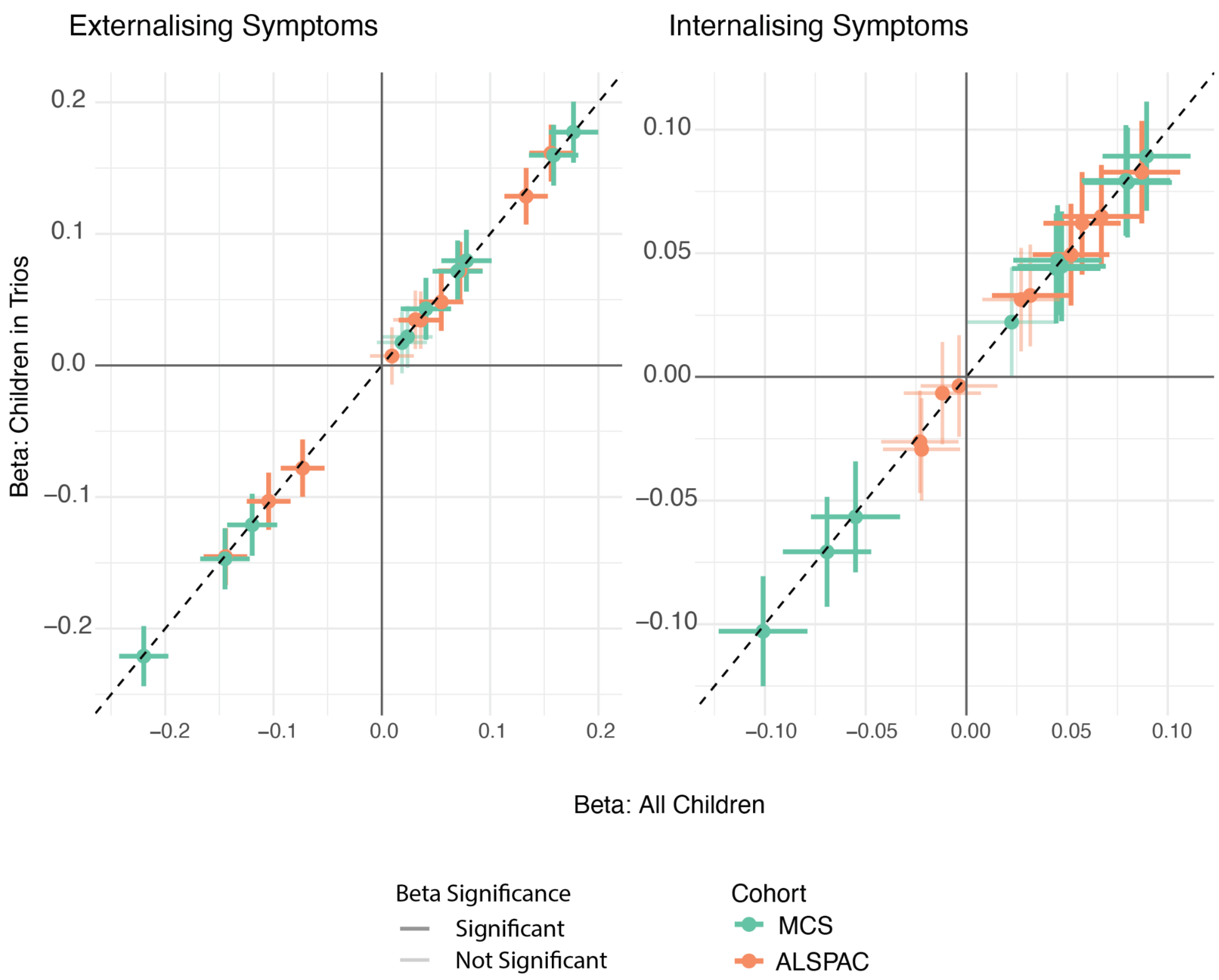

Supplementary Figure 7. Comparison of standardised effect estimates for associations between polygenic indices (PGIs) and internalising and externalising symptoms for models estimated using all children versus children in trios. Standardised effect sizes (beta) and 95% confidence intervals are shown for the main effect of each polygenic index, estimated per standard deviation change in symptom score. Children in trios included imputed trios following Mendelian imputation of missing parental genotypes for children genotyped as duos. Dashed line indicates the y=x axis.

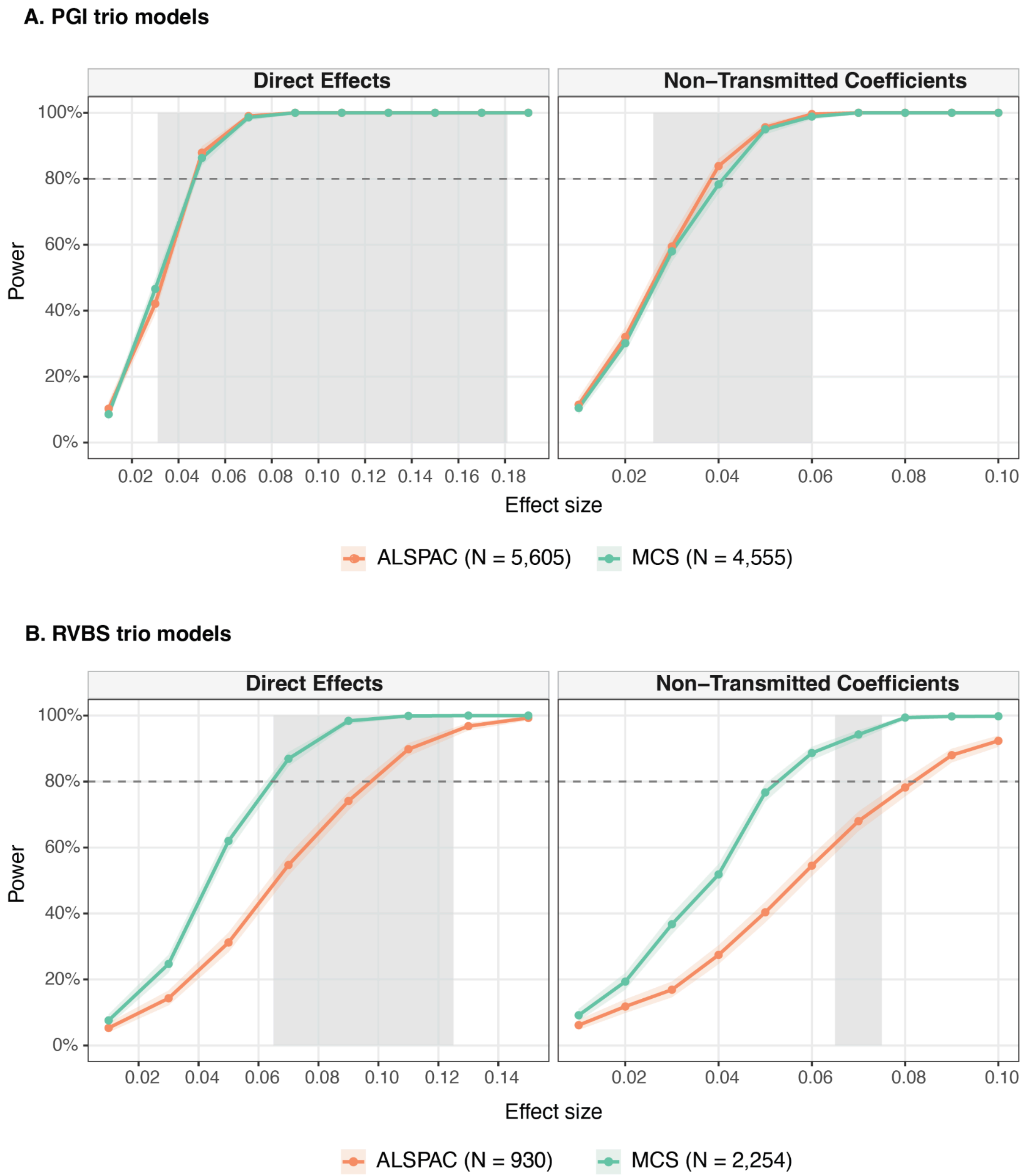

Supplementary Figure 8. Power curves for post-hoc power analysis for trio models. Power curves show the proportion of 1,000 simulations in which the genetic effect (child direct effect or parental NTC) reached nominal significance ($p<0.05$) in the trio models across a range of effect sizes in ALSPAC and MCS. Grey shaded regions indicate the range of nominally significant effect sizes observed in our analyses for direct effect estimates and parental NTCs (Supplementary Tables 3,4, and 5). The dashed horizontal line denotes 80% power. Ribbons show 95% confidence intervals. (A) Power for direct effects and parental non-transmitted coefficients (NTCs) in the PGI trio models (ALSPAC N=5,605; MCS N=4,555). (B) Power for direct effects and NTCs in the RVBS trio models (ALSPAC N=930; MCS N=2,254).

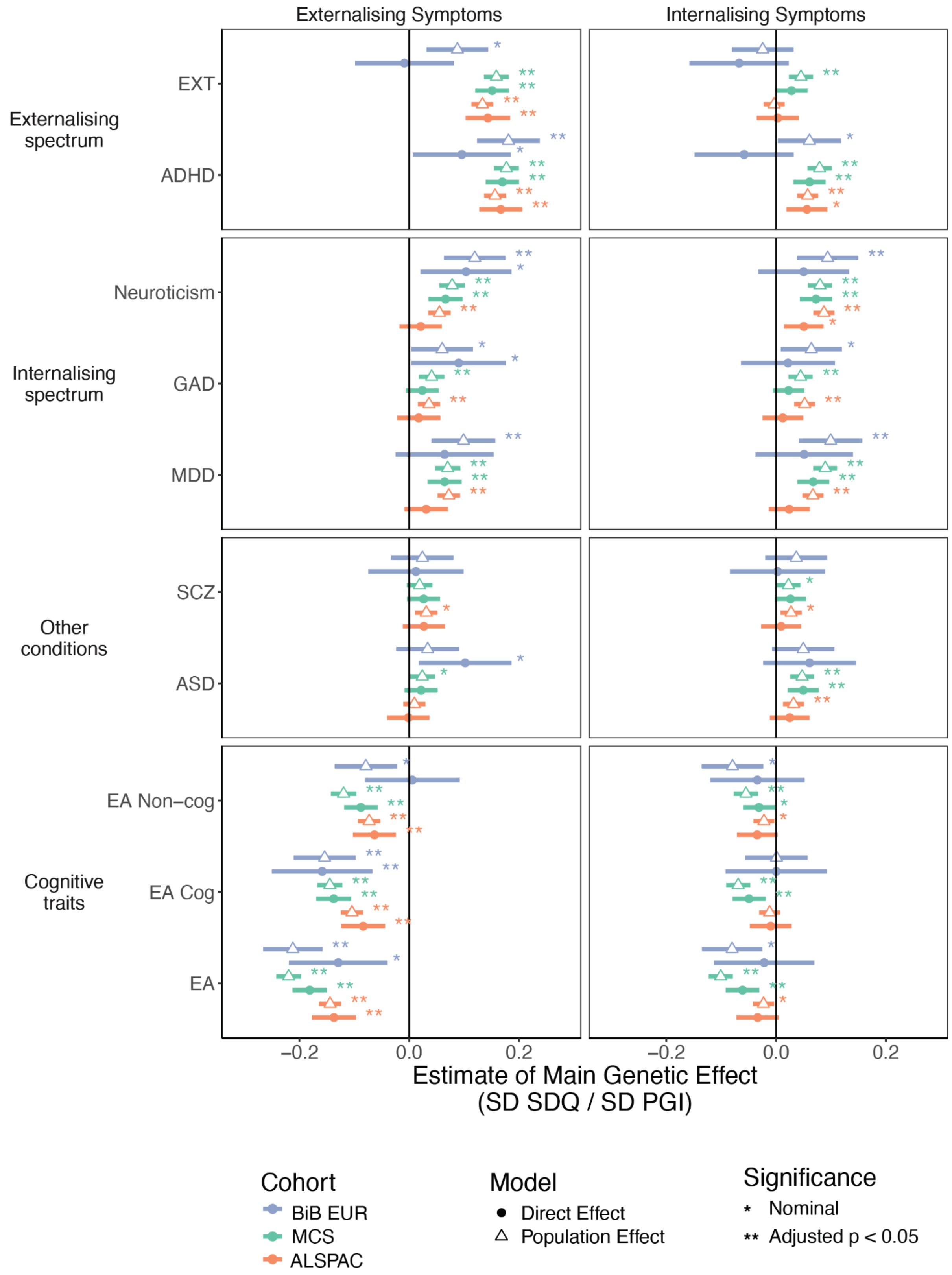

Supplementary Figure 9. Associations between polygenic indices (PGIs) and childhood and adolescent externalising and internalising symptoms. Standardised effects and 95% confidence intervals estimated for the main effects of the genetic score per standard deviation change in symptom score. Population effect sizes were estimated in the full sample (triangle) and direct genetic

effects were estimated in models controlling for parental genetic scores (shaded circles). Effect size estimates for MCS and ALSPAC were estimated using linear mixed effect models; estimates for BiB were calculated using linear models with the earliest parent-reported SDQ measurement as the outcome. ADHD: Attention-deficit hyperactivity disorder; ASD: Autism spectrum disorder; EA: educational attainment; EA Cog: cognitive component of EA; EA Non-cog: non-cognitive component of EA; EXT: externalising behaviours; GAD: Generalised anxiety disorder; MDD: Major depressive disorder; SCZ: schizophrenia.

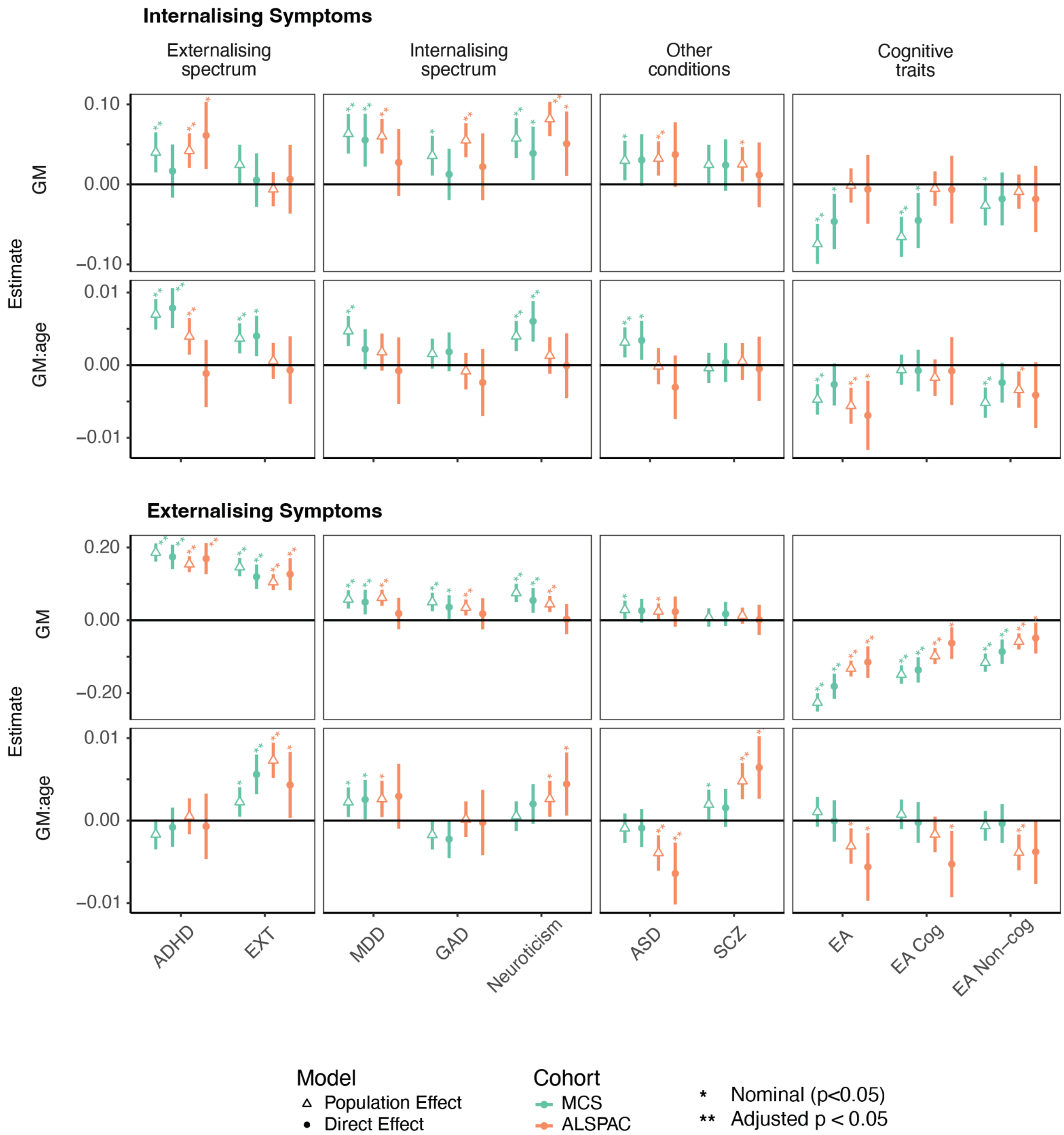

Supplementary Figure 10. Association between PGIs and internalising and externalising symptoms across ages for linear mixed effect models estimated with a PGI:age interaction effect. Standardised effect sizes and 95% confidence intervals are shown from linear mixed-effects models including a PGI-by-age interaction term. Estimates are presented for both the main effect of the genetic measure (GM) and the GM × age interaction, per standard deviation change in symptom score. Population effect sizes were estimated in the full sample (triangle) and direct genetic effects were estimated in models controlling for parental genetic scores (shaded circles). ADHD: Attention-deficit hyperactivity disorder; ASD: Autism spectrum disorder; EA: educational attainment; EA Cog: cognitive component of EA; EA Non-cog: non-cognitive component of EA; EXT: externalising behaviours; GAD: Generalised anxiety disorder; MDD: Major depressive disorder; SCZ: schizophrenia.

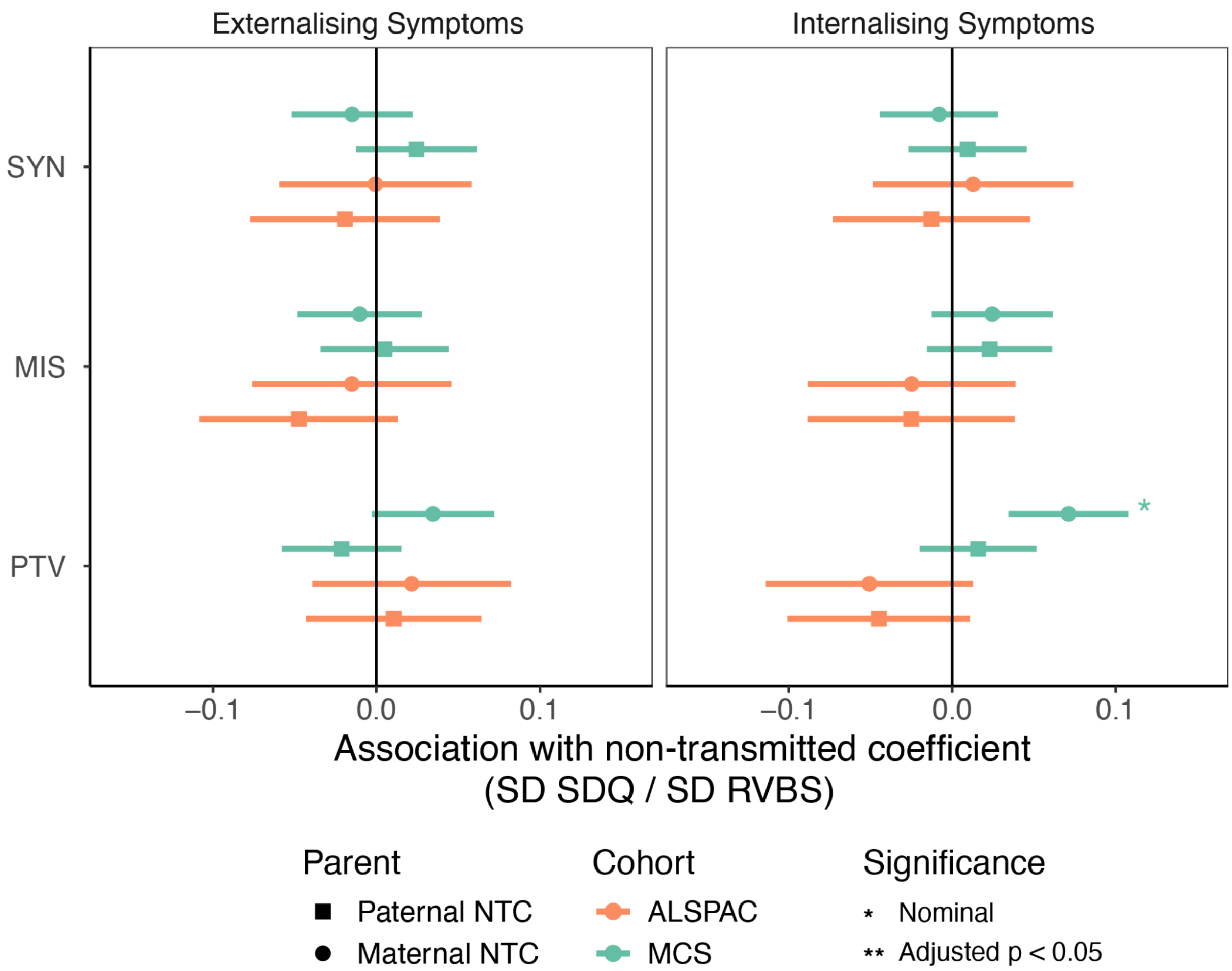

Supplementary Figure 11. Effect size estimates for parental non-transmitted coefficients (NTCs) for rare variant burden scores (RVBS) estimated using trio models in MCS and ALSPAC. Error bars represent the 95% confidence interval for the estimate. MIS: missense; PTV: protein truncating variant; SYN: synonymous.

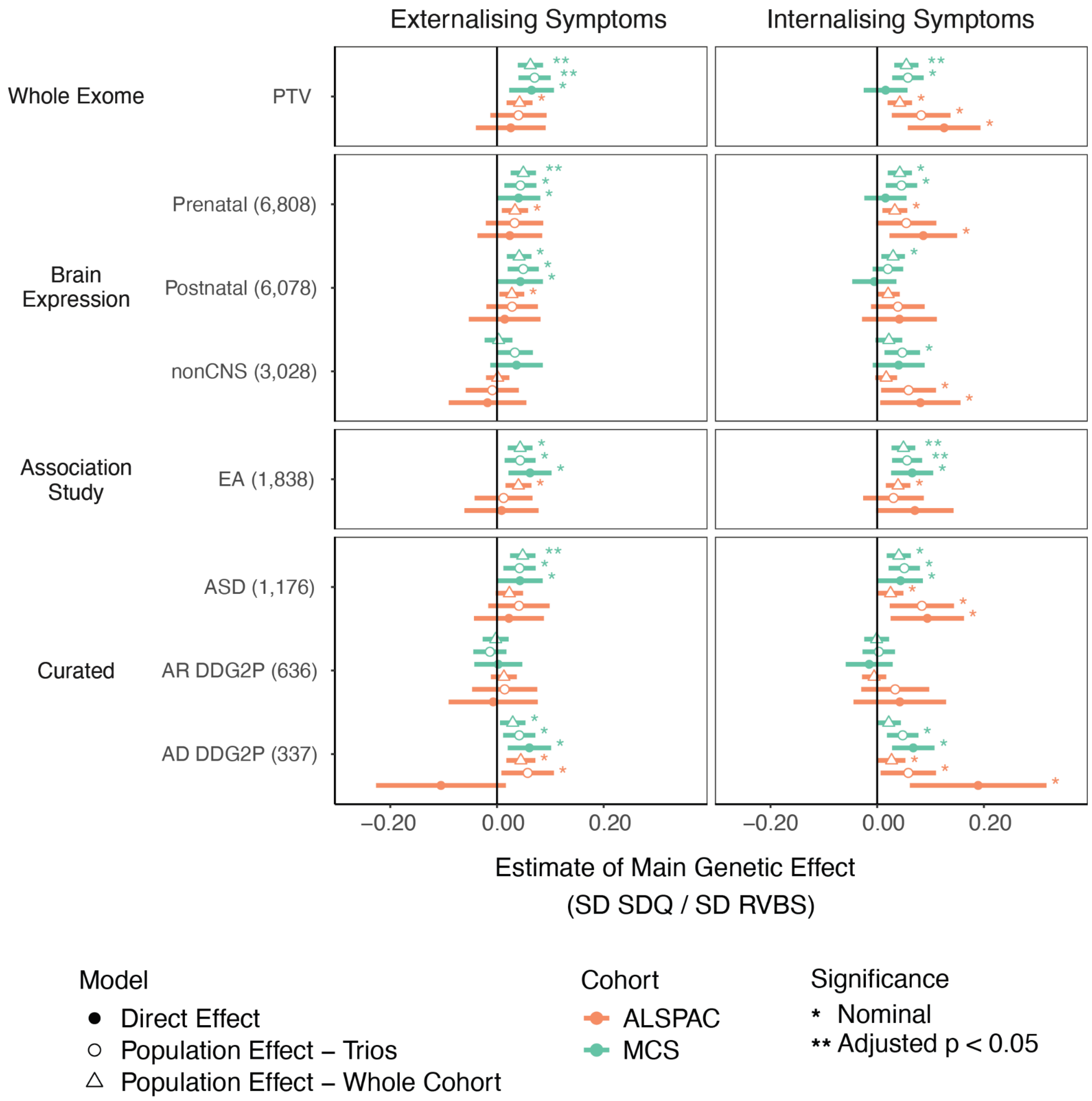

Supplementary Figure 12. Associations between gene set rare variant burden scores (RVBS) for protein-truncating variants and childhood and adolescent externalising and internalising symptoms. Standardised effects (in standard deviations of SDQ score) and 95% confidence intervals estimated for the main effects of the RVBS per standard deviation change in symptom score for RVBS calculated for protein-truncating variants in selected gene sets. Population effect sizes were estimated in the full sample (circle) and in a subset of children with parental RVBS (triangle). Shaded circles illustrate direct effect estimates from models controlling for parental genetic scores. AD DDG2P: autosomal dominant developmental disorder genes; AR DDG2P: autosomal recessive developmental disorder genes; ASD: autism spectrum disorders; CNS: central nervous system; EA: educational attainment; PTV: protein truncating variant.

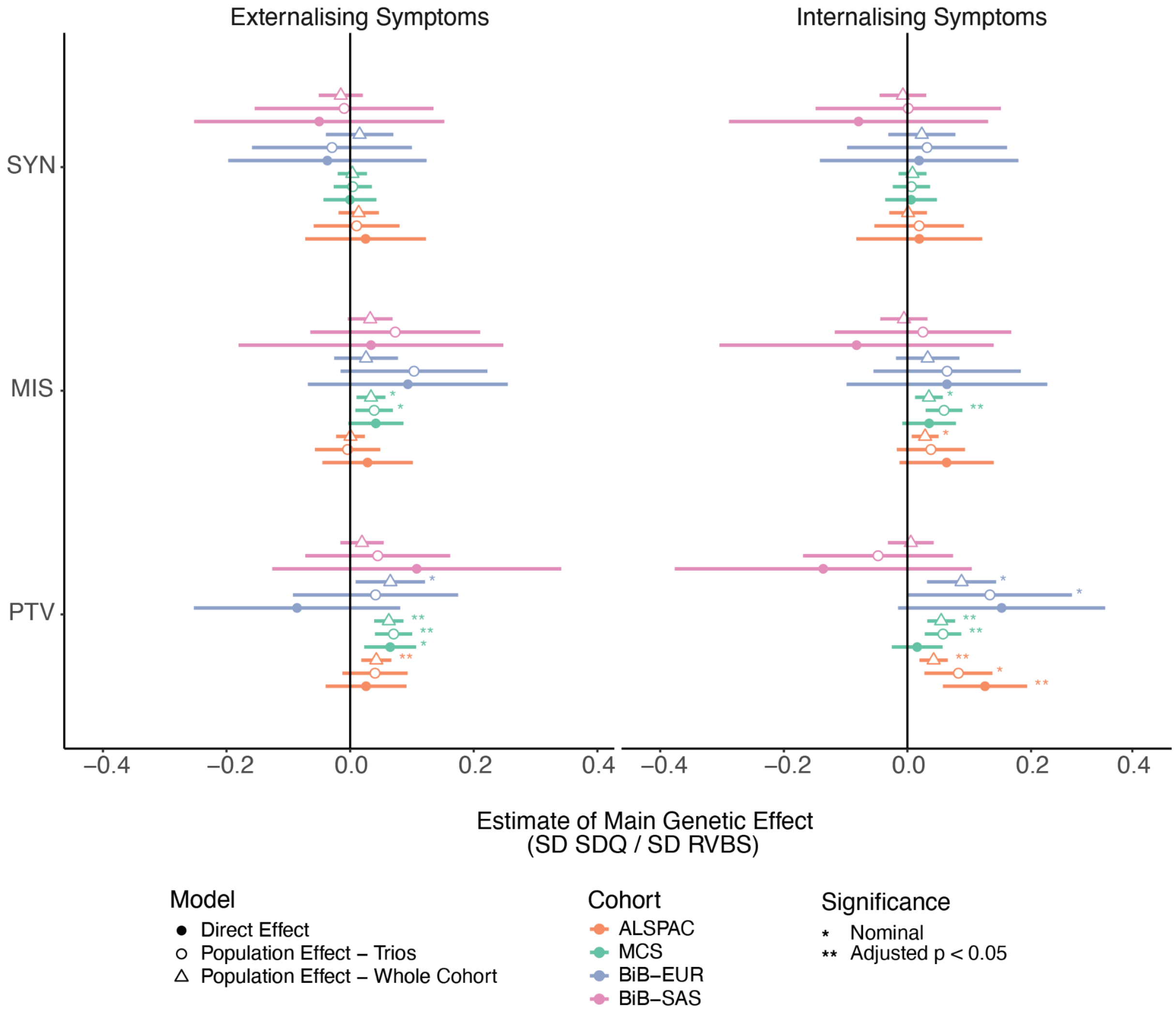

Supplementary Figure 13. Associations between rare variant burden scores (RVBS) and childhood and adolescent externalising and internalising symptoms. Standardised effects and 95% confidence intervals estimated for the main effects of the RVBS per standard deviation change in symptom score for RVBS calculated with three different consequence classes. Population effect sizes were estimated in the full sample (circle) and in a subset of children with parental RVBS (triangle). Shaded circles illustrate direct effect estimates from models controlling for parental genetic scores. Effect size estimates for MCS and ALSPAC were estimated using linear mixed effect models; estimates for BiB were calculated using linear models with the earliest parent-reported SDQ measurement as the outcome. MIS: missense; PTV: protein truncating variant; SYN: synonymous.

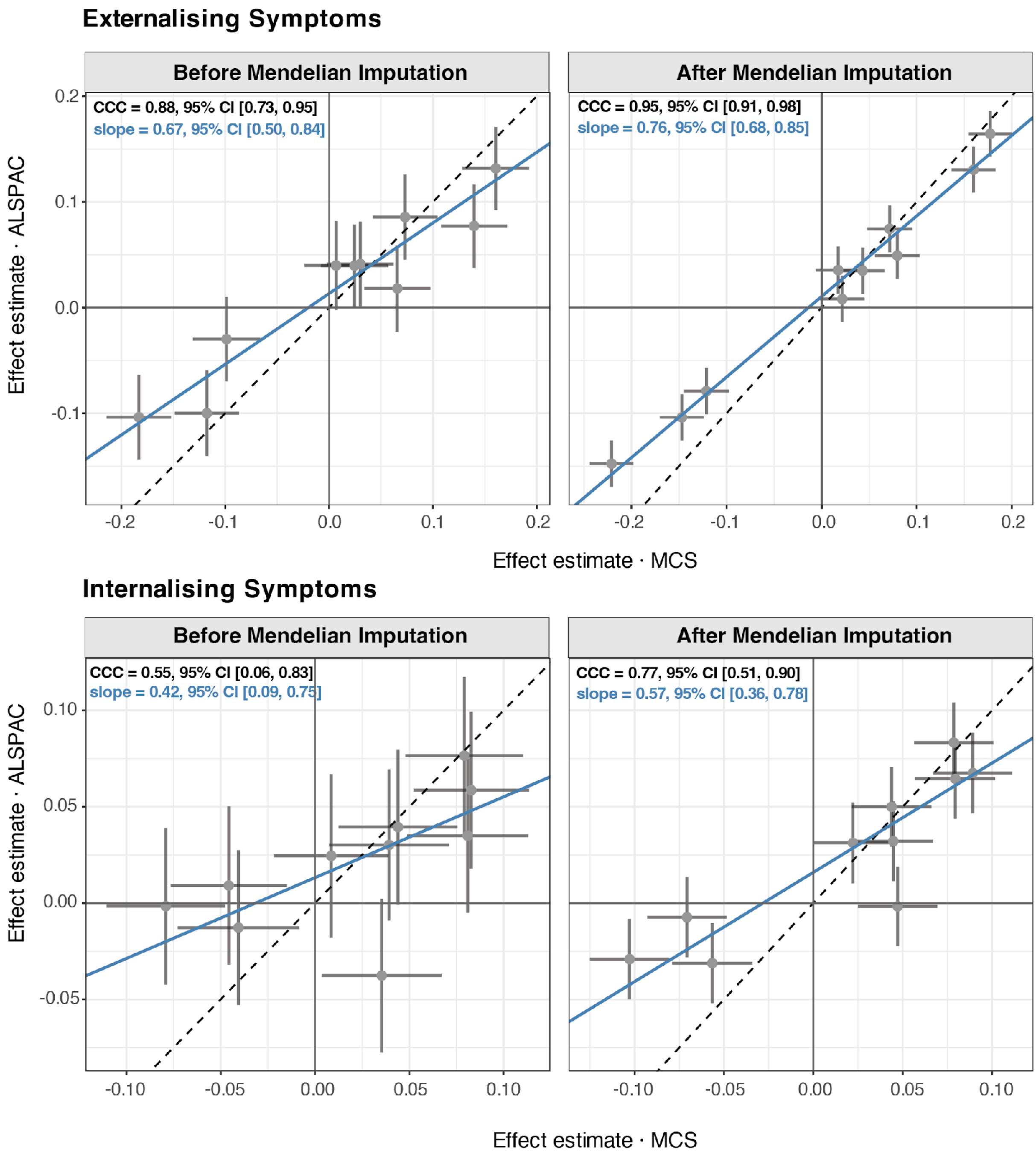

Supplementary Figure 14. Comparison of population-level associations between ALSPAC and MCS before and after Mendelian imputation of missing parental genotypes. Population-level associations were estimated for children genotyped as complete trios ("Before" Mendelian imputation - both parents genotyped) and children from observed and imputed trios ("After" Mendelian imputation - children genotyped as duos). Points represent standardised effect estimates, and error bars show 95% confidence intervals. Blue line represents the Deming regression fit. Dashed line indicates the y=x axis. CCC is Lin's concordance correlation coefficient.

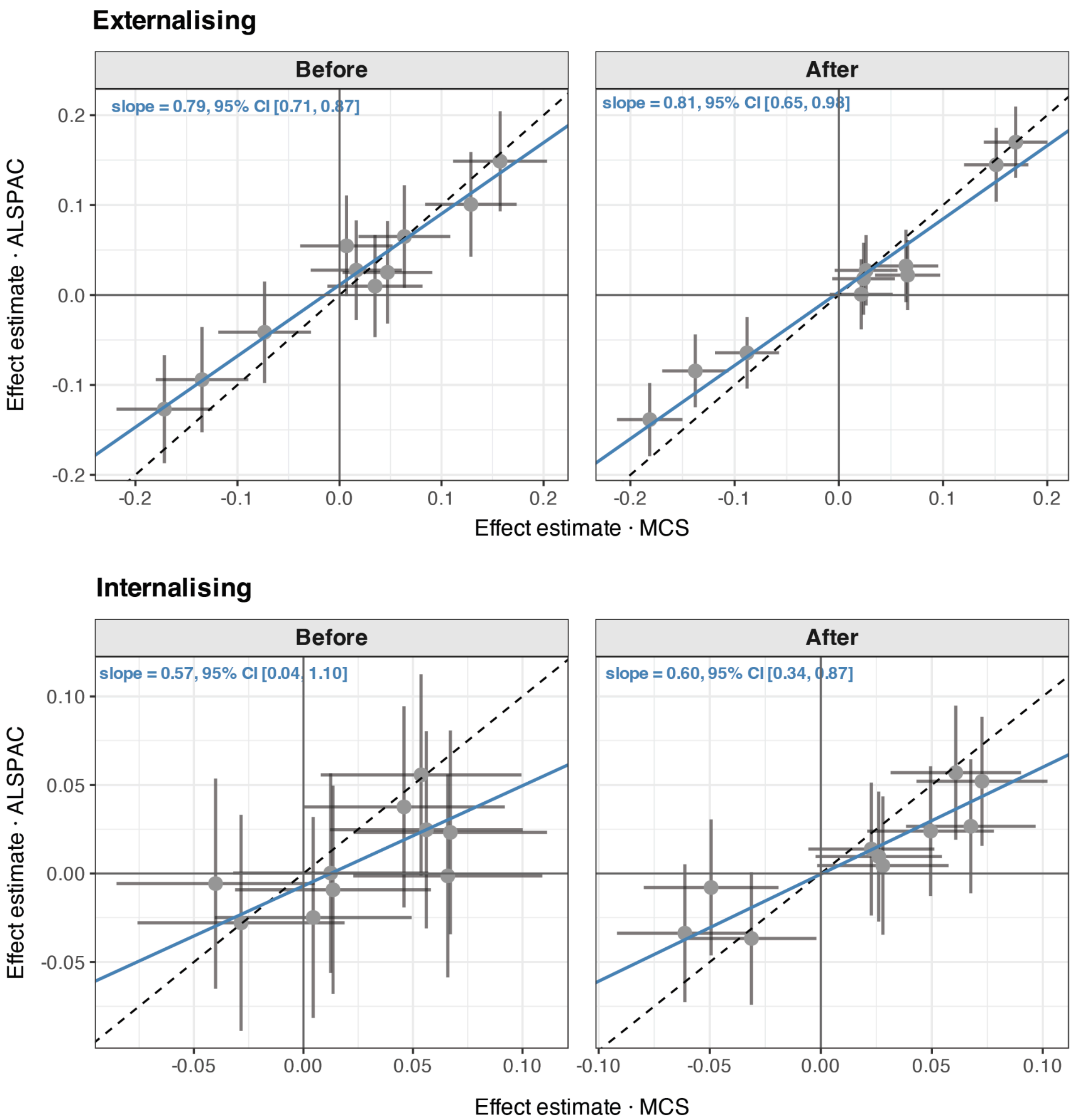

Supplementary Figure 15. Comparison of direct genetic effect estimates between ALSPAC and MCS before and after Mendelian imputation of missing parental genotypes. Direct genetic effect associations were estimated using trio models for children genotyped as complete trios ("Before" Mendelian imputation - both parents genotyped) and children from observed and imputed trios ("After" Mendelian imputation - children genotyped as duos). Points represent standardised effect estimates, and error bars show 95% confidence intervals. Blue line represents the Deming regression fit. Dashed line indicates the y=x axis. CCC is Lin's concordance correlation coefficient.

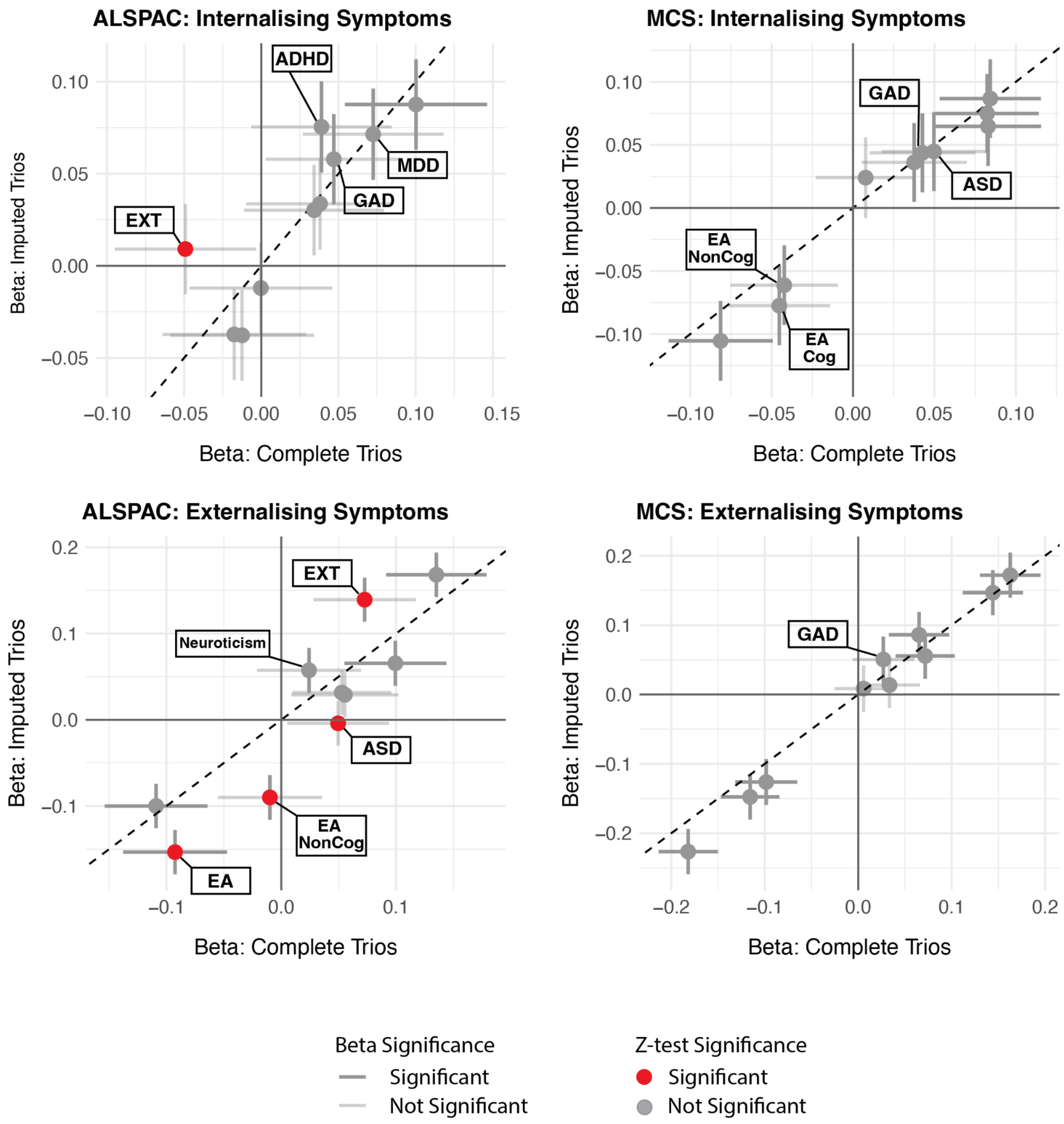

Supplementary Figure 16. Comparison of standardised effect estimates for population-level associations between polygenic indices (PGIs) and internalising and externalising symptoms using complete versus imputed trios. Population-level associations were estimated for children genotyped as complete trios (both parents genotyped) and children from observed and imputed trios (including children genotyped as duos, the missing parental genotype was imputed using Mendelian imputation). Points represent standardised effect estimates, and error bars show 95% confidence intervals. The opacity of the error bars reflects the significance of the effect estimate. Circles are shaded red where Z-tests indicate a significant difference between estimates derived from complete and imputed trios. Dashed line indicates the y=x axis.

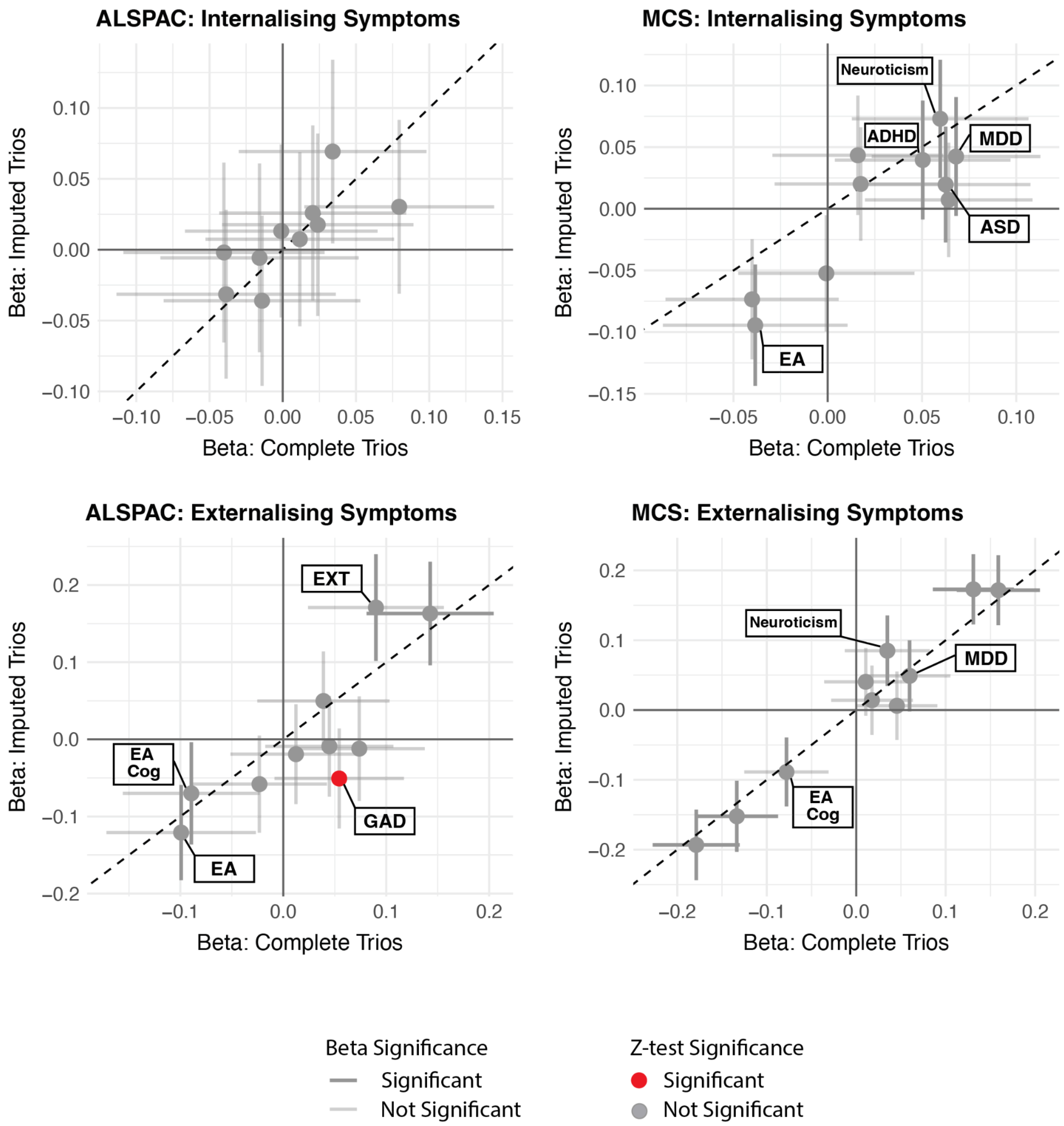

Supplementary Figure 17. Comparison of standardised direct genetic effect estimates for associations between polygenic indices (PGIs) and internalising and externalising symptoms using complete versus imputed trios. Direct genetic effect associations were estimated using trio models for children genotyped as complete trios (both parents genotyped) and imputed trios (children genotyped as duos, the missing parental genotype was imputed using Mendelian imputation). Points represent standardised effect estimates, and error bars show 95% confidence intervals. The opacity of the error bars reflects the significance of the effect estimate. Circles are shaded red where Z-tests indicate a significant difference between estimates derived from complete and imputed trios. Dashed line indicates the y=x axis.

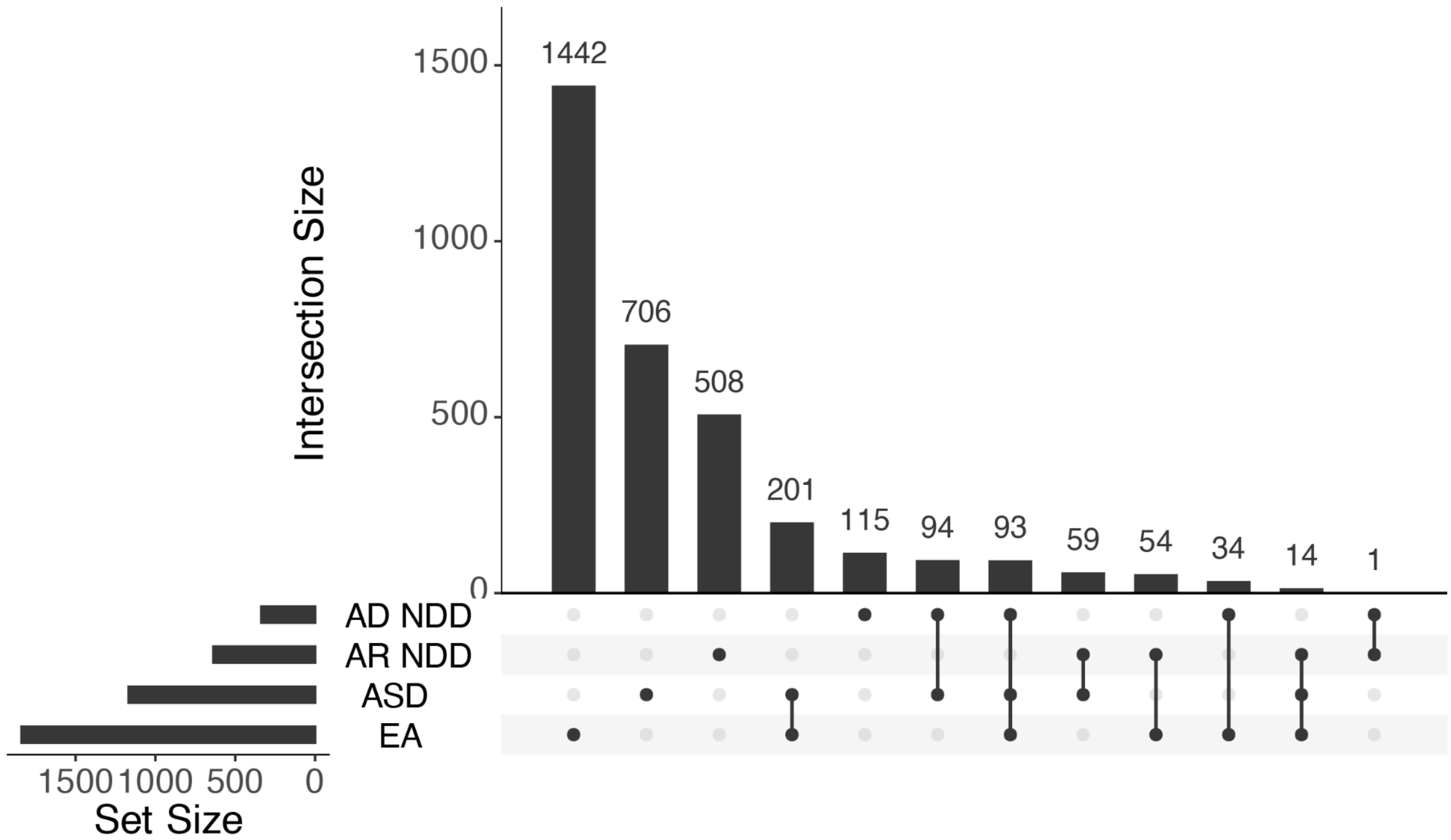

Supplementary Figure 18. Overlap across the gene sets used to calculate gene set-specific rare variant burden scores. An UpSet plot showing the overlap of protein-coding genes from each gene set. AD DDG2P: autosomal dominant developmental disorder genes; AR DDG2P: autosomal recessive developmental disorder genes; ASD: autism spectrum disorders; EA: educational attainment.

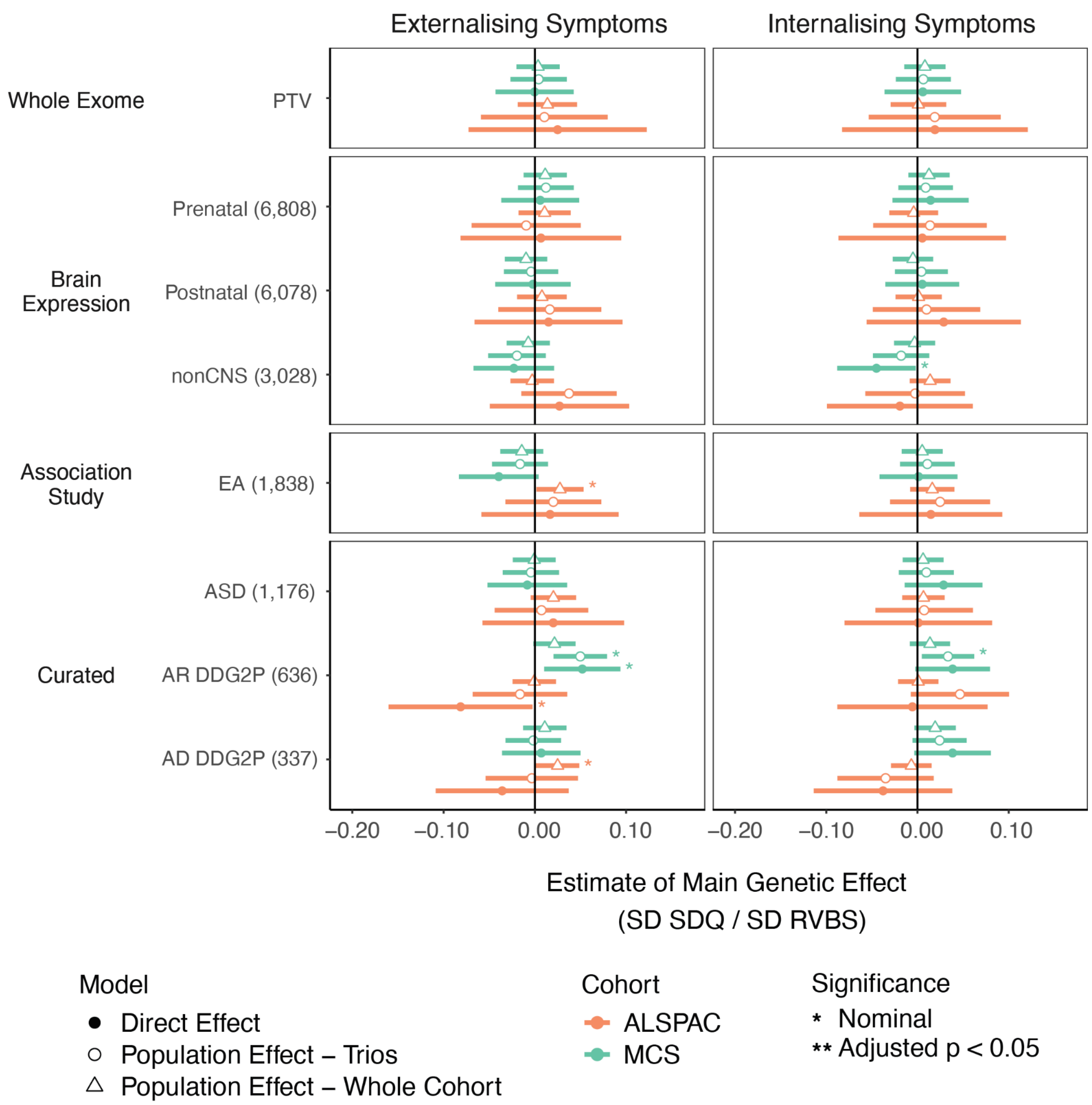

Supplementary Figure 19. Associations between gene-set rare variant burden scores (RVBS) for synonymous variants and childhood and adolescent externalising and internalising symptoms. Standardised effects (in standard deviations of SDQ score) and 95% confidence intervals estimated for the main effects of the RVBS per standard deviation change in symptom score for RVBS calculated for synonymous variants in selected gene sets. Population effect sizes were estimated in the full sample (circle) and in a subset of children with parental RVBS (triangle). Shaded circles illustrate direct effect estimates from models controlling for parental genetic scores. AD DDG2P: autosomal dominant developmental disorder genes; AR DDG2P: autosomal recessive developmental disorder genes; ASD: autism spectrum disorders; CNS: central nervous system; EA: educational attainment; SYN: synonymous variant.

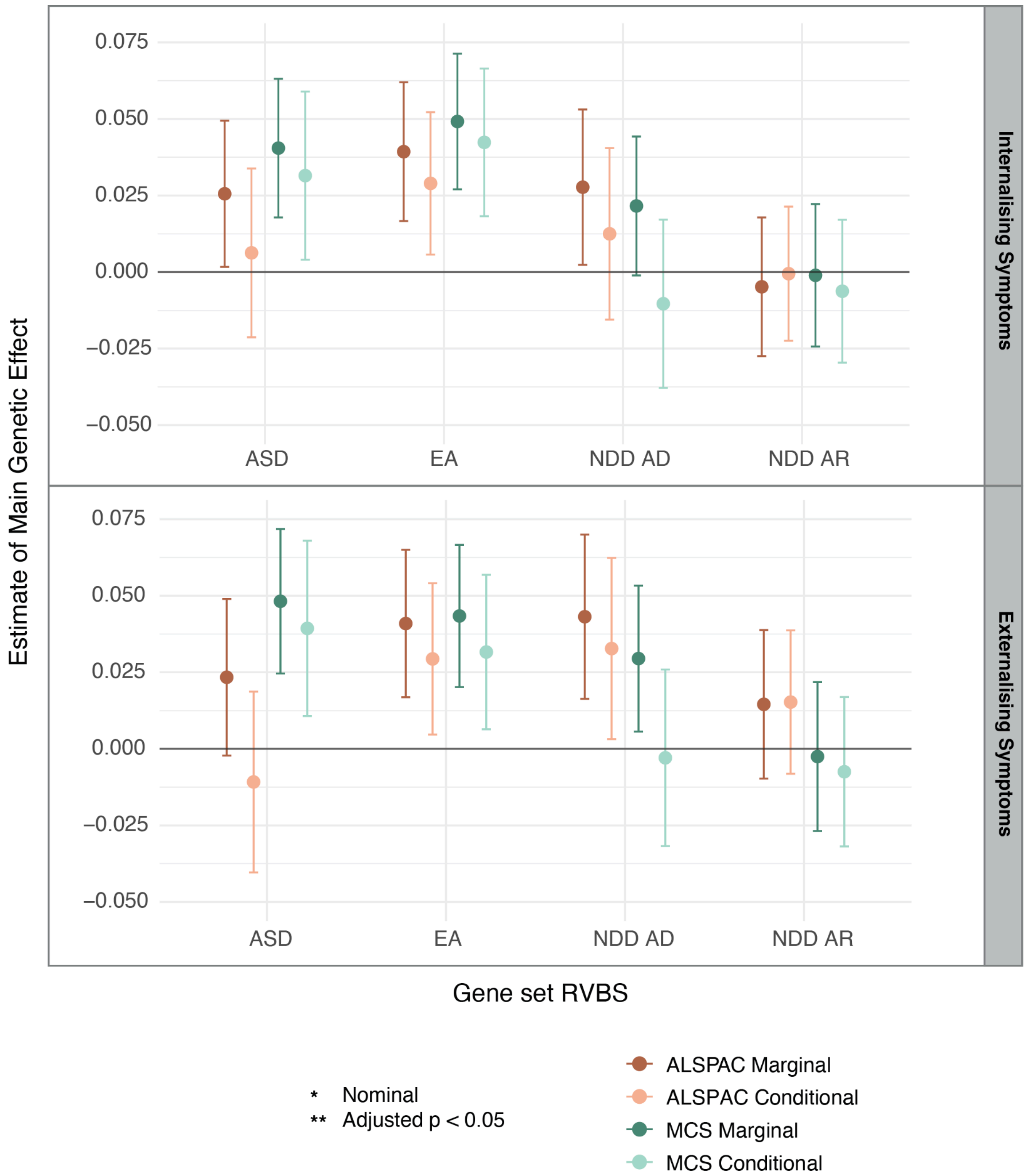

Supplementary Figure 20. Associations with gene set rare variant burden scores (RVBS) in marginal vs. conditional models. Standardised effect sizes and 95% confidence intervals are shown for the main effects of each gene set-specific RVBS, estimated using two models per cohort: (1) marginal models including only the indicated RVBS and standard covariates, and (2) conditional models including all gene set RVBS as predictors. Models were fit in all children with exome sequence data and SDQ scores (population effect; whole cohort). AD DDG2P: autosomal dominant developmental disorder genes; AR DDG2P: autosomal recessive developmental disorder genes; ASD: autism spectrum disorders; EA: educational attainment; PTV: protein truncating variant.

Supplementary Note 1: Post-hoc power analysis for trio models

To better contextualise the results of the trio models, we conducted a post-hoc power analysis. For each cohort and genetic measure type (PGI and RVBS), we simulated longitudinal data matching the variance components, sample size, and timepoint distribution of our analytical sample, with child and parental genetic measures jointly drawn from a theoretical covariance matrix reflecting Mendelian transmission and assortative mating. Power was estimated at the nominal significance threshold ($p<0.05$) across a range of effect sizes for direct effects, maternal NTCs, and paternal NTCs, with 1,000 simulations per condition.

For PGI direct effects, power exceeded 80% at $\beta \approx 0.05$ in both ALSPAC and MCS (Supplementary Figure 8), encompassing the full range of significant direct effects observed in our analyses (β = 0.05 to 0.18). For PGI NTCs, power exceeded 80% at $\beta \approx 0.04$ in both cohorts. The significant NTCs in our results, all maternal NTCs in MCS for EA-related PGIs ($\beta = -0.055$ to -0.060)(Supplementary Table 4), fall in a region of high power, while the smaller nominally significant NTCs (β=0.03 to 0.04) sit closer to the boundary of detectability. Power was estimated using the nominal significance threshold ($p<0.05$) rather than the Benjamini-Yekutieli adjusted threshold used to determine significance in our primary analyses. Power at the adjusted threshold would be lower than the values reported here. However, the small number of NTC associations observed at even the nominal threshold (Supplementary Table 4) is consistent with limited evidence for true NTC associations for PGIs.

For the RVBS analyses, power was substantially lower than for the PGI analyses, due, at least in part, to the smaller trio sample sizes with exome sequence data. Power for RVBS direct effects exceeded 80% at $\beta \approx 0.11$ in ALSPAC and $\beta \approx 0.07$ in MCS (Supplementary Figure 8). Given the limited power for the RVBS analyses, the single significant RVBS direct effect we report ($RVBS_{PTV}$ on internalising symptoms in ALSPAC, β=0.13) should be interpreted as preliminary evidence requiring replication. Power for RVBS NTCs exceeded 80% at $\beta \approx 0.08$ in ALSPAC and $\beta \approx 0.05$ in MCS. No significant RVBS NTCs were observed in our analyses (Supplementary Figure 11), but as research on indirect genetic effects of rare variants is limited, the magnitudes at which such effects might plausibly occur are not yet well established. Larger sequenced trio cohorts are needed for within-family analyses of rare variant associations, and we suggest this as a direction for future research.

Overall, these results indicate that our PGI trio models had reasonable statistical power to detect NTCs of modest magnitude ($\beta \geq 0.04$). The absence of significant NTC associations for the externalising behaviour and ADHD PGIs is therefore unlikely to reflect insufficient statistical power, supporting the interpretation that these PGIs primarily capture direct genetic effects, consistent with previous PGI-based studies[22–25]. For internalising symptoms, the nominally significant maternal NTCs we observed for neuroticism, depression, and anxiety PGIs (β=0.03 to 0.04) sit closer to or below the β=0.04 power threshold, therefore these associations should be treated as preliminary evidence requiring replication in larger samples. For RVBS analyses, the lower power available, means that null RVBS findings should be interpreted with appropriate caution.

Supplementary Note 2: Gene set associations

To test whether the observed associations between $RVBS_{PTV}$ and mental health symptoms are explained by PTVs in specific gene sets, we reestimated linear mixed effect models using gene set-specific RVBS. We considered sets of genes in which rare variants are known to cause dominant or recessive developmental disorders (the DDG2P list[16]), a set of genes robustly linked to autism through rare/de novo variant associations (the SFARI list[17]), and a third set of genes prioritised using DEPICT from the EA GWAS[26]. We additionally considered gene sets defined by brain expression, including genes assigned to co-expressed modules enriched for expression in the prenatal brain, postnatal brain, or not expressed in the brain, based on spatiotemporal transcriptomic data[18]. After correction for multiple testing, no gene set-specific RVBS were significantly associated with internalising or externalising symptoms in both cohorts, although the association between $RVBS_{PTV}$ for genes preferentially expressed in the prenatal brain and externalising symptoms remained significant in population models in MCS (p-adj=0.02)(Supplementary Figure 12).

We observed several associations consistent across ALSPAC and MCS at a nominal level of significance ($p<0.05$; p-adj>0.05)(Supplementary Figure 12). Burden of PTVs in the autism spectrum disorder gene set was associated with childhood internalising symptoms in both the population and trio models. $RVBS_{PTV}$ for autosomal dominant developmental disorder genes was associated with externalising symptoms in population models. Notably, we observed no significant association for $RVBS_{PTV}$ in the autosomal recessive developmental disorder genes, presumably reflecting the fact that these variants are very rare and so most individuals in these population cohorts are only heterozygous carriers. $RVBS_{PTV}$ in genes associated with educational attainment showed a nominally significant association in population models estimated using all children for both symptom domains, though this association attenuated in ALSPAC when restricting to children in exome-sequenced trios.

The analyses of brain-expression gene sets showed that rare variant associations with symptom scores were more consistently observed for variants in genes expressed in the brain than for genes not expressed in the brain, although most associations did not survive multiple-testing correction. In both cohorts, $RVBS_{PTV}$ in the prenatal gene set was associated with both symptom domains in population models estimated using all children at a nominal level of significance ($p<0.05$; p-adj>0.05), while associations in exome-sequenced trios and direct genetic effects were consistent in direction across the cohorts but not in statistical significance. $RVBS_{PTV}$ restricted to genes expressed in the postnatal brain also showed several nominal associations ($p<0.05$; p-adj>0.05), specifically for externalising symptoms in MCS and for externalising symptoms in the full ALSPAC sample. In contrast, $RVBS_{PTV}$ for genes not expressed in the brain showed no associations with externalising symptoms in either cohort, and only nominal associations with internalising symptoms in trio-based analyses and in ALSPAC. Together, these results suggest that the exome-wide rare variant signal may be primarily explained by variants in genes expressed in the brain.

We also observed a few nominal associations between certain synonymous variant gene set RVBS and symptom scores (Supplementary Figure 19). These associations were inconsistent in direction and magnitude across cohorts and are therefore unlikely to reflect true biological signal. The absence of associations with the exome-wide synonymous RVBS

(Figure 4) further suggests that associations observed in the gene set synonymous RVBS analysis likely reflect noise due to limited power.

To explore the relative contribution of each gene set $RVBS_{PTV}$, we estimated a conditional model including gene set RVBS for educational attainment, developmental disorder, and autism spectrum disorder. Across both cohorts, effect size estimates for individual gene sets were attenuated relative to those in the marginal models, and most associations were no longer significant (Supplementary Figure 20), likely due to overlap between these gene sets (Supplementary Figure 18). The association between the educational attainment $RVBS_{PTV}$ and both symptom domains remained nominally significant in the conditional models, indicating that this association was not fully explained by overlap with the other gene sets. Although this finding suggests that deleterious rare variants in genes associated with educational attainment may be of particular relevance to early-life mental health, the EA gene set is the largest gene set and thus, the nominally significant associations in the joint model should be interpreted with caution.

Supplementary Note 3: Comparison of main genetic effects between ALSPAC and MCS

To assess the agreement of main PGI effect estimates between cohorts before and after Mendelian imputation of missing parental genotypes, we estimated weighted Deming regression models. These were estimated separately for each symptom domain using population effect estimates from trio-based models (Population Effect - Trios) and direct genetic effect estimates (see Supplementary Methods). Lin's concordance correlation coefficient (CCC) was also calculated to provide a single summary measure of agreement.

For both symptom domains, the Deming regression intercept was not significantly different from zero before imputation, indicating that constant bias was minimal. For externalising symptoms, agreement between ALSPAC and MCS was already high when only observed trios were analysed (Deming slope: population effect 0.67, [95% CI: 0.50-0.84] and direct effect 0.79, [95% CI: 0.71-0.87]) (Supplementary Figure 14-15; Supplementary Table 14). Agreement improved further using imputed genotyped trios (Deming slope: population effect 0.76, [95% CI: 0.68-0.85] and direct effect 0.81, [95% CI: 0.65-0.98]), with the largest improvement observed for population effect models (Lin's CCC: 0.88 before imputation versus 0.95 after imputation) (Supplementary Table 14).

For internalising symptoms, agreement was more modest for models estimated using observed genotyped trios only (Deming slope: population effect 0.42 [95 % CI: 0.09–0.75] and direct effect 0.57 [95 % CI: 0.04–1.10]) (Supplementary Figures 16-17; Supplementary Table 14). Imputing missing parental genotypes improved agreement for both population and direct effect estimates (Deming slope: population effect 0.57 [95 % CI: 0.36–0.78] and direct effect 0.60 [95 % CI: 0.34–0.87]), with the largest relative gain seen for population effect estimates (Lin's CCC: 0.56 before imputation versus 0.80 after imputation).

Overall, imputation increased the consistency of PGI effect estimates between cohorts by reducing proportional bias, with the greatest improvements observed for internalising symptoms and population effect estimates.

References

1. Boyd A, Golding J, Macleod J, Lawlor DA, Fraser A, Henderson J, et al. Cohort Profile: the 'children of the 90s'--the index offspring of the Avon Longitudinal Study of Parents and Children. Int J Epidemiol. 2013;42:111–127.
2. Fraser A, Macdonald-Wallis C, Tilling K, Boyd A, Golding J, Davey Smith G, et al. Cohort Profile: the Avon Longitudinal Study of Parents and Children: ALSPAC mothers cohort. Int J Epidemiol. 2013;42:97–110.
3. Northstone K, Ben Shlomo Y, Teyhan A, Hill A, Groom A, Mumme M, et al. The Avon Longitudinal Study of Parents and children ALSPAC G0 Partners: A cohort profile. Wellcome Open Res. 2023;8:37.
4. Koko M, Fabian L, Popov I, Eberhardt RY, Zakharov G, Huang QQ, et al. Exome sequencing of UK birth cohorts. Wellcome Open Res. 2024;9:390.
5. Manichaikul A, Mychaleckyj JC, Rich SS, Daly K, Sale M, Chen W-M. Robust relationship inference in genome-wide association studies. Bioinformatics. 2010;26:2867–2873.
6. 1000 Genomes Project Consortium, Auton A, Brooks LD, Durbin RM, Garrison EP, Kang HM, et al. A global reference for human genetic variation. Nature. 2015;526:68–74.
7. Patterson N, Price AL, Reich D. Population structure and eigenanalysis. PLoS Genet. 2006;2:e190.
8. Price AL, Weale ME, Patterson N, Myers SR, Need AC, Shianna KV, et al. Long-range LD can confound genome scans in admixed populations. Am J Hum Genet. 2008;83:132–135; author reply 135–139.
9. Taliun D, Harris DN, Kessler MD, Carlson J, Szpiech ZA, Torres R, et al. Sequencing of 53,831 diverse genomes from the NHLBI TOPMed Program. Nature. 2021;590:290–299.
10. Das S, Forer L, Schönherr S, Sidore C, Locke AE, Kwong A, et al. Next-generation genotype imputation service and methods. Nat Genet. 2016;48:1284–1287.
11. McCarthy S, Das S, Kretzschmar W, Delaneau O, Wood AR, Teumer A, et al. A reference panel of 64,976 haplotypes for genotype imputation. Nat Genet. 2016;48:1279–1283.
12. Fitzsimons E, Moulton V, Hughes DA, Neaves S, Ho K, Hemani G, et al. Collection of genetic data at scale for a nationally representative population: the UK Millennium Cohort Study. Longit Life Course Stud. 2021;13:169–187.
13. Huang QQ, Wigdor EM, Malawsky DS, Campbell P, Samocha KE, Chundru VK, et al. Examining the role of common variants in rare neurodevelopmental conditions. Nature. 2024:1–8.
14. Arciero E, Dogra SA, Malawsky DS, Mezzavilla M, Tsismentzoglou T, Huang QQ, et al. Fine-scale population structure and demographic history of British Pakistanis. Nat Commun. 2021;12:7189.
15. Lee JJ, Wedow R, Okbay A, Kong E, Maghzian O, Zacher M, et al. Gene discovery and polygenic prediction from a genome-wide association study of educational attainment in 1.1 million individuals. Nat Genet. 2018;50:1112–1121.
16. Yates TM, Ansari M, Thompson L, Hunt SE, Uhalte EC, Hobson RJ, et al. Curating genomic disease-gene relationships with Gene2Phenotype (G2P). Genome Med. 2024;16:127.
17. Banerjee-Basu S, Packer A. SFARI Gene: an evolving database for the autism research

community. Dis Model Mech. 2010;3:133–135.
18. Li M, Santpere G, Imamura Kawasawa Y, Evgrafov OV, Gulden FO, Pochareddy S, et al. Integrative functional genomic analysis of human brain development and neuropsychiatric risks. Science. 2018;362:eaat7615.
19. Green P, MacLeod CJ. SIMR: an R package for power analysis of generalized linear mixed models by simulation. Methods Ecol Evol. 2016;7:493–498.
20. Yengo L, Robinson MR, Keller MC, Kemper KE, Yang Y, Trzaskowski M, et al. Imprint of assortative mating on the human genome. Nat Hum Behav. 2018;2:948–954.
21. Border R, O'Rourke S, de Candia T, Goddard ME, Visscher PM, Yengo L, et al. Assortative mating biases marker-based heritability estimators. Nat Commun. 2022;13:660.
22. Frach L, Barkhuizen W, Allegrini AG, Ask H, Hannigan LJ, Corfield EC, et al. Examining intergenerational risk factors for conduct problems using polygenic scores in the Norwegian Mother, Father and Child Cohort Study. Mol Psychiatry. 2024;29:951–961.
23. Martin J, Wray M, Agha SS, Lewis KJS, Anney RJL, O'Donovan MC, et al. Investigating direct and indirect genetic effects in attention-deficit/hyperactivity disorder using parent-offspring trios. Biol Psychiatry. 2023;93:37–44.
24. Tanksley PT, Brislin SJ, Wertz J, de Vlaming R, Courchesne-Krak NS, Mallard TT, et al. Do polygenic indices capture 'direct' effects on child externalizing behavior problems? Within-family analyses in two longitudinal birth cohorts. Clin Psychol Sci. 2025;13:316–331.
25. van der Laan CM, van de Weijer SGA, Pool R, Hottenga J-J, van Beijsterveldt TCEM, Willemsen G, et al. Direct and indirect genetic effects on aggression. Biol Psychiatry Glob Open Sci. 2023;3:958–968.
26. Lee JJ, Wedow R, Okbay A, Kong E, Maghzian O, Zacher M, et al. Gene discovery and polygenic prediction from a 1.1-million-person GWAS of educational attainment. Nat Genet. 2018;50:1112.
